# Phylogenomic analysis of UDP glycosyltransferase 1 multigene family in *Linum usitatissimum* identified genes with varied expression patterns

**DOI:** 10.1186/1471-2164-13-175

**Published:** 2012-05-08

**Authors:** Vitthal T Barvkar, Varsha C Pardeshi, Sandip M Kale, Narendra Y Kadoo, Vidya S Gupta

**Affiliations:** 1 Plant Molecular Biology Group, Biochemical Sciences Division, National Chemical Laboratory, Pune, 411008, India

## Abstract

**Background:**

The glycosylation process, catalyzed by ubiquitous glycosyltransferase (GT) family enzymes, is a prevalent modification of plant secondary metabolites that regulates various functions such as hormone homeostasis, detoxification of xenobiotics and biosynthesis and storage of secondary metabolites. Flax (*Linum usitatissimum* L.) is a commercially grown oilseed crop, important because of its essential fatty acids and health promoting lignans. Identification and characterization of UDP glycosyltransferase (UGT) genes from flax could provide valuable basic information about this important gene family and help to explain the seed specific glycosylated metabolite accumulation and other processes in plants. Plant genome sequencing projects are useful to discover complexity within this gene family and also pave way for the development of functional genomics approaches.

**Results:**

Taking advantage of the newly assembled draft genome sequence of flax, we identified 137 UDP glycosyltransferase (UGT) genes from flax using a conserved signature motif. Phylogenetic analysis of these protein sequences clustered them into 14 major groups (A-N). Expression patterns of these genes were investigated using publicly available expressed sequence tag (EST), microarray data and reverse transcription quantitative real time PCR (RT-qPCR). Seventy-three per cent of these genes (100 out of 137) showed expression evidence in 15 tissues examined and indicated varied expression profiles. The RT-qPCR results of 10 selected genes were also coherent with the digital expression analysis. Interestingly, five duplicated UGT genes were identified, which showed differential expression in various tissues. Of the seven intron loss/gain positions detected, two intron positions were conserved among most of the UGTs, although a clear relationship about the evolution of these genes could not be established. Comparison of the flax UGTs with orthologs from four other sequenced dicot genomes indicated that seven UGTs were flax diverged.

**Conclusions:**

Flax has a large number of UGT genes including few flax diverged ones. Phylogenetic analysis and expression profiles of these genes identified tissue and condition specific repertoire of UGT genes from this crop. This study would facilitate precise selection of candidate genes and their further characterization of substrate specificities and *in planta* functions.

## Background

Flax or linseed (*Linum usitatissimum* L.) is one of the earliest domesticated crops. It is a self-pollinating diploid species cultivated as a source of fibre, oil and medicinal compounds. Historically it has been used as a model for developmental studies and has a different evolutionary history than other model plants like Arabidopsis [1]. Among plant foods, flaxseed has the highest contents of the essential omega-3 fatty acid, alpha-linolenic acid (ALA) [2] and bioactive phenolic compounds such as lignans, predominantly secoisolariciresinol diglucoside (SDG) [3], phenolic acids and flavonoids [4]. ALA dampens inflammatory reactions, thereby reducing a risk of heart attack or stroke; while lignans are strong antioxidants inhibiting breast and prostate cancers. Given the economic and health benefits of these bioactive compounds, it would be useful to comprehensively analyze the genes involved in their biosynthesis. In plants, glycosylation represents the last step in the biosynthesis of numerous natural compounds like terpenes, phenylpropanoids, cyanogenic glucosides and glucosinolates. It is an important modification that alters their activity, sub-cellular location and modulates their chemical properties, such as solubility and stability, which are important for their *in planta* functions [5].

The glycosylation process is catalyzed by glycosyltransferase enzymes (GTs), which are highly divergent, polyphyletic and belong to a multigene family found in all living organisms [6]. GTs from diverse species have been classified into 92 families based on the amino acid sequence similarities, catalytic mechanisms and the presence of conserved sequence motifs (http://www.cazy.org/GlycosylTransferases.html). Among these, the glycosyltransferase family 1 is the largest family, the enzymes of which generally catalyze transfer of the glycosyl group from nucleoside diphosphate-activated sugars (e.g., UDP-sugars) to a diverse array of substrates, including hormones, secondary metabolites and xenobiotics such as pesticides and herbicides [5,7]. The plant UGT enzymes are characterized by a unique, well-conserved sequence of 44 amino acid residues designated as the plant secondary product glycosyltransferases (PSPG) box [8] and a catalytic mechanism that inverts the anomeric configuration of a transferred sugar [9].

The GT family 1 has been extensively studied in various plants species, as well as in humans. In mammals, UGTs coordinate the activity of signal molecules such as steroid hormones and detoxify xenobiotic compounds taken up from the environment [10]. Polymorphisms among these UGTs have been shown to be associated with increased susceptibility to certain diseases in humans [11]. Studies in model plants have shown that the plant genomes contain a great diversity of gene sequences predicted to be involved in glycosylation [12,13]. The occurrence of a wide range of glycosylated products in flax [3] suggests the presence of a large number of UGTs. The availability of the flax genome sequence (http://linum.ca), tissue specific ESTs (http://www.ncbi.nlm.nih.gov/nuccore?term=Linum%20usitatissimum) and microarray expression dataset [14] (http://www.ncbi.nlm.nih.gov/projects/geo/) of flax provide an opportunity to analyze the diversity of expressed glycosyltransferase family genes in this economically important oilseed crop.

In this study, we identified 137 UGT genes from flax, which were clustered into 14 phylogenetically distinct groups. Their expression patterns were analyzed using 15 tissue specific EST libraries available at the NCBI as well as the publicly available microarray expression data, which indicated their differential expression in various flax tissues. This digital expression analysis was further supported by RT-qPCR for ten selected genes. Seven flax diverged UGTs were identified from the families 75, 79 and 94, which indicated diversification of flax UGTs as compared to those of four other sequenced dicots, *viz*., *Ricinus communis, Populus trichocarpa*, *Vitis vinifera* and *Arabidopsis thaliana*.

## Results

### Identification of flax UGT genes

BlastP search against the 47,912 flax gene models (http://linum.ca) using the conserved PSPG box sequence resulted in the identification of 179 scaffolds. Family 1 UGTs usually utilize low molecular weight compounds as acceptor substrates and UDP-sugars as donors [12] and commonly possess a carboxy terminal consensus sequence (PSPG box) believed to be involved in binding to the UDP moiety of the sugar nucleotide donor [9,15]. Taking these characteristics into account, 137 sequences (GenBank accession numbers JN088282-JN088418) having lengths of 375–530 amino acids and 0–2 introns were selected and subjected to phylogenetic and digital expression analysis. In order to confirm the open reading frame (ORF) sequence of these genes, 11 genes expressed in seed tissue were randomly selected, isolated using PCR, cloned and sequenced, which revealed that they were 100% identical to the putative UGT gene sequences identified.

### Phylogenetic analysis

**Figure 1:**
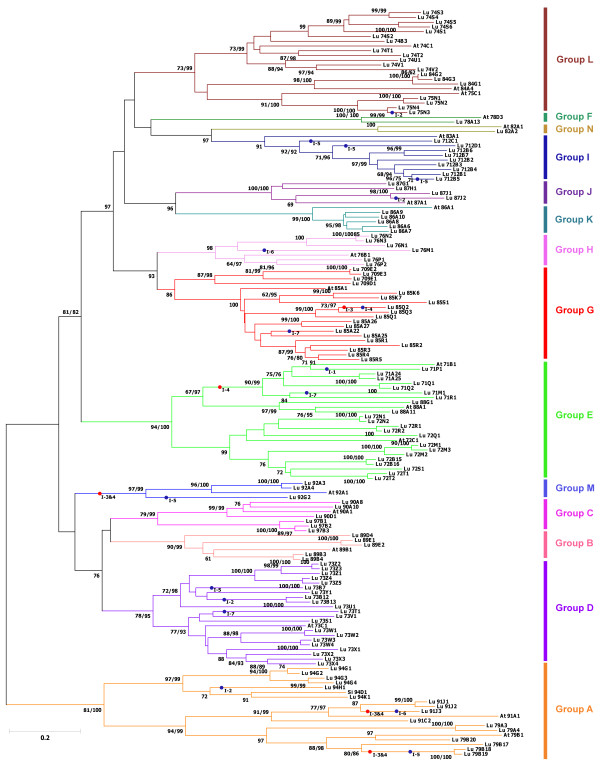
**Phylogenetic analysis of the*Linum usitatissimum*UGT family genes.** The tree was derived by neighbour‒joining distance analysis of alignable regions comprising ~60% of the UGT sequences using MEGA5. Bootstrap values over 60% are indicated at the nodes, with the *number* on the *left* for neighbour-joining and *right* for parsimony methods. Hypothetical positions of intron gain and loss are indicated by *dots* followed by intron number and it is assumed that introns 3 and 4 were gained prior to diversification of flax UGTs (see Figure 2). Postulated intron gains are indicated by blue dots and intron losses by red dots. Eighteen Arabidopsis and one Sesame UGT sequences from each UGT family were included in the analysis (Accession numbers given in Additional file 2).

All the identified putative UGT genes were classified as per the recommendations of the UGT Nomenclature Committee [6] (Additional file 1). As expected, the PSPG signature motif was present in all the UGT sequences and the overall sequence similarity among them varied substantially from 36% to 98% (Additional file 2). A total of 409 amino acid positions (60.41% of the sequences) were aligned for all the genes analyzed and used to construct a phylogenetic tree. Fourteen major groups (A-N) were defined by both the neighbour-joining (NJ) and parsimony methods with high bootstrap supports (>85) (Figure 1). The tree topology and grouping of the UGTs were similar as described for the Arabidopsis UGT genes [16], e.g. group L consists of the UGTs belonging to the families 74, 75 and 84. However, in four groups, A, C, G and I, sequences from additional UGT families were observed *viz.* LuUGT94, LuUGT97, LuUGT709 and LuUGT712, respectively. The number of genes (1–22) as well as the sequence diversity varied considerably within each group (Additional file 2).

### Detection of orthologs and duplicated genes

The orthologs of flax UGTs identified in the four selected dicots are listed in the Additional file 3. Of the 137 sequences, orthologs were identified for 130 UGTs from at least one of the four dicots. However, for 72 sequences, orthologs were identified from all the four species. The maximum number of orthologs (125) was identified in case of *Vitis vinifera*, while the lowest of 80 orthologs were detected in case of *Arabidopsis thaliana*. Seven flax diverged UGTs were identified (*LuUGT94G1*, *LuUGT94G2*, *LuUGT94G3*, *LuUGT94G4*, *LuUGT94H1*, *LuUGT75N3* and *LuUGT79A4*) and 22 gene duplication events with sequence similarity of ~90% were observed (Additional file 4).

### Analysis of intron gain/loss events

**Figure 2:**
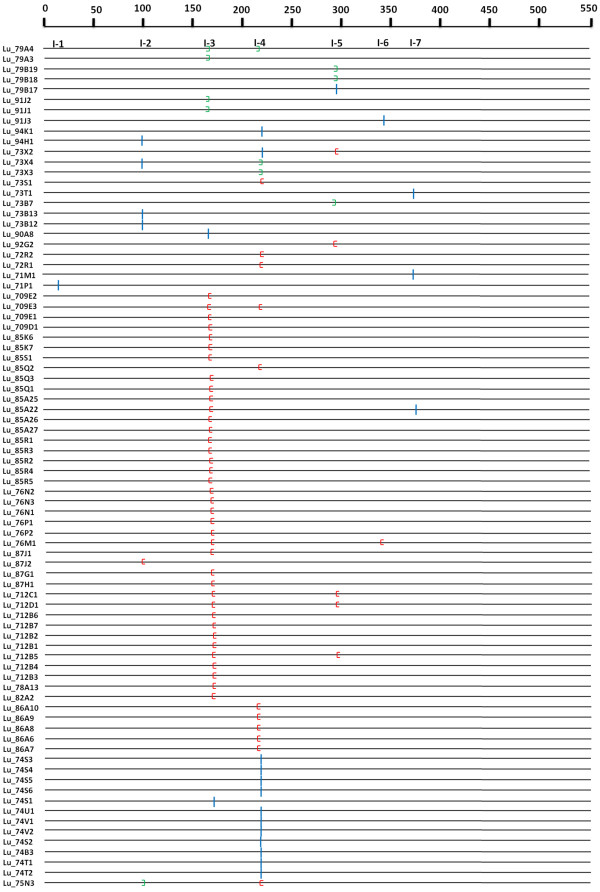
**Distribution of introns among 82 UGT genes of *Linum usitatissimum.*** The introns are mapped and numbered to the alignment of their amino acid sequences. It is hypothesized that the introns 3 and 4 were gained prior to diversification of flax UGTs and the gain and loss of other introns in the genes within a phylogenetic group are indicated by the colored mark. The numbers on the top of the map show the intron insertion number occurred on each gene. Intron phases are indicated by blue bar, red open bracket and green close bracket for zero, one and two, respectively.

Among the 137 sequences, 55 were intron less, while 72 and 10 had one and two introns each, respectively (Additional file 1). Total 92 introns were detected in the 137 UGTs, with an average of 0.67 intron per gene. Seven independent intron insertion events were observed when the intron positions were compared with the sequence relationship predicted by the phylogenetic analysis (Figure 2). An intron was considered conserved if its position in a particular sequence was within 40–45 amino acids of its mean recorded position across the sequences (for complete sequence alignment see Additional file 5: Figure S1). Two conserved introns (intron 3 and intron 4; Additional file 1) were identified, of which intron 3 was observed in 44 UGTs belonging to the A, C and F-J phylogenetic groups, while intron 4 was observed in 27 UGTs belonging to the D, E, K and L phylogenetic groups. *LuUGT79A4* from group A and *LuUGT709E3* from group G both had the conserved introns. Alternatively, group M showed absence of both the conserved introns, while *LuUGT92G2* from group M showed gain of intron 5. Within the members of groups F-J and N, intron 3 was predominant, except in *LuUGT85Q2* and *LuUGT87J2*. In comparison, the members of groups K and L had intron 4, while only one member of L group (*LuUGT74S1*) showed the presence of intron 3. All other introns were either found only within a single restricted group of closely related sequences or in only a single gene. Group B members were intron less.

Many sequences showed loss of the conserved introns and gain of other introns. For example, within group A, three members from family 79 and one member from family 91 (*LuUGT91J3*) showed loss of conserved introns 3 and 4, and gain of introns 5 and 6, respectively. Similarly, within group D, four members of family 73 lost conserved intron 4 and few members gained introns 2, 5 and 7. Likewise, in group E, all the members of the family 71 showed loss of conserved intron 4 while gain of introns 1, 7 and 8 in few members.

Most of the conserved introns were either in phase 1 (49 genes) or phase 0 (15 genes) (Additional file 1). The intron sizes of flax UGTs ranged from 65 bp to 2258 bp with an average of 406 bp for both the introns. About 28% of the flax UGT introns were in the size range of 65–99 bp (Additional file 6: Figure S2).

In Arabidopsis, 37 out of 88 UGT genes contained introns while, three genes had two introns. By comparing the intron positions with sequence relationships predicted by phylogenetic analysis, a minimum of nine independent intron insertion events appear to have happened in the course of UGT evolution in Arabidopsis. Intron 2 was found to be widespread and oldest intron and was present in all of the 23 UGT sequences in groups F–K in Arabidopsis [12]. Similarly in flax, the introns 3 and 4 have been found in most members of the groups F-J and K respectively and could be considered as the oldest introns.

### Expression analysis of flax UGT genes using EST data

Expression of the identified UGT genes was analyzed using the available EST and microarray data of flax. Of the 137 genes, 100 genes showed expression evidence based on either or both the datasets. Among these, 85 genes (62.04%) were expressed based on the EST data; while the microarray data indicated expression evidence for 60 genes (43.79%) (Additional file 7). Similarly for 45 genes, the expression evidence was present in both the datasets. Further, the ESTs from various flax tissues were mapped onto the 137 flax UGT gene models to estimate their gene expression levels. This analysis identified that a total of 325 ESTs mapped to 85 flax UGT sequences with an average of 3.82 ESTs per gene. The frequency of ESTs varied greatly from 1 to 54 per UGT gene model. Among the various tissue types, flower (FL, 18.46%) and seed coat at torpedo stage (TC, 15.69%) had the largest number of highly expressed genes, while globular embryo (GE) stage had the lowest (2, 0.61%) number of expressed genes.

The highest number of ESTs (91) were mapped to 13 sequences of group G, followed by 69 ESTs mapping to 15 members of group E. On the contrary, only one EST was mapped to a single group N member. On an average, the highest of 7.00 ESTs were mapped per UGT sequence of family G, followed by 4.60 ESTs per gene of family E. The percentage of the genes expressed per phylogenetic group or family varied from 28% to 100% (Additional file 7). Among all the genes expressed, *LuUGT85Q2* and *LuUGT74S1* showed the highest expression in flower (FL) and seed coat at torpedo stage (TC), respectively (Additional file 7).

### Expression analysis of flax UGT genes using microarray data

**Figure 3:**
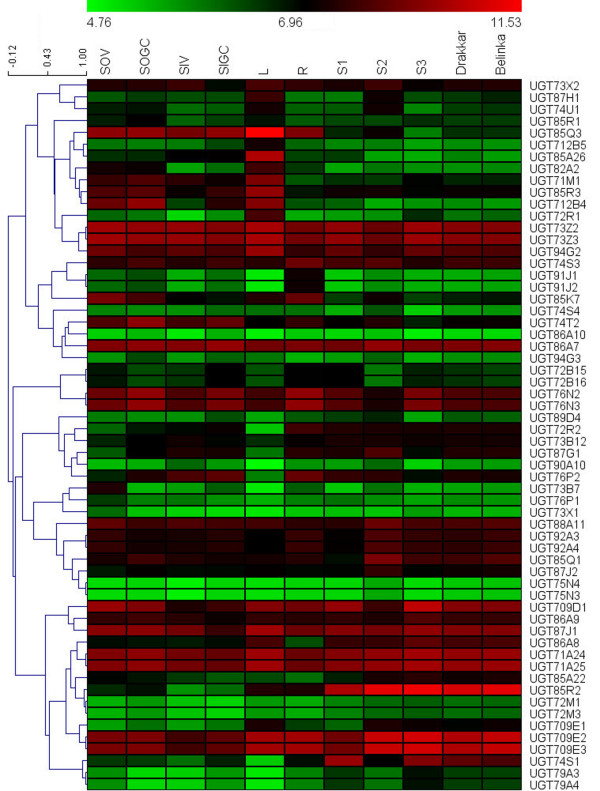
**Expression levels for flax UGT genes in various tissues by microarray analysis.** The RMA‒normalized, average log2 signal values of flax UGTs in various tissues and seed developmental stages (listed at the top of heat map) were used for construction of the heat map. The left side of the heat map shows hierarchical clustering based on Pearson correlation matrix. The colour scale (representing log2 signal values) is shown at the top. Microarray data from stem outer tissues; vegetative stage (SOV), stem outer tissues, green capsule stage (SOGC), stem inner tissues; vegetative stage (SIV), stem inner tissues; green capsule stage (SIGC), leaves (L), roots (R), seeds, 10–15 DAF (S1), seeds, 20–30 DAF (S2) and seeds, 40–50 DAF (S3) were used for constructing the expression heat map.

In addition to the sequence based expression analysis method, we also used publicly available microarray data (http://www.ncbi.nlm.nih.gov/geo/query/acc.cgi?acc=GSE21868) under the platform GSE21868, which profiles expression patterns for various flax tissues and seed developmental stages, *viz*., roots (R), leaves (L), stem outer tissues: vegetative stage (SOV), stem outer tissues: green capsule stage (SOGC), stem inner tissues: vegetative stage (SIV), stem inner tissues: green capsule stage (SIGC), seeds: 10–15 days after flowering (DAF) (S1), seeds: 20–30 DAF (S2) and seeds: 40–50 DAF (S3) [14]. We used the Robust Multichip Average (RMA) -normalized, averaged gene-level log2 values for expression evidence of UGTs to construct a heat map (Figure 3). Hierarchical clustering with Pearson correlation matrix highlighted co-expression of specific gene family members in specific tissue types. Only 60 of the 137 (43.79%) flax UGTs represented on the array showed expression evidence (Additional file 7). Three genes were highly expressed in seed stages S2 and S3 (averaged gene-level log2 value: *LuUGT85R2* (11.11 and 11.30), *LuUGT709E2* (10.57 and 10.76), and *LuUGT709E3* (10.57 and 10.76), respectively; while one gene (*LuUGT85Q3*, averaged gene-level log2 value: 11.53) showed the highest expression in leaf tissue (Figure 3). The number of genes having higher expression in different tissues (averaged gene-level log2 values >6.96) varied from 14 (S1) to 24 (SOGC) (Additional file 7). Among the different tissues, SOGC had the largest number of highly expressed genes, while S3 had the lowest (23%) (Additional file 7). Surprisingly, the two contrasting varieties, Drakkar and Belinka did not show any difference in the expression of these 60 UGTs (Figure 3).

### Expression profiling using RT-qPCR

The RT-qPCR is currently the most accurate method for detecting differential gene expression. The 12 tissue types selected for UGT expression profiling cover all plant parts and seed developmental stages from fertilization to seed maturation. *Eukaryotic translation initiation factor 5A* (ETIF5A GenBank ID GR508912) was selected as a reference gene after confirming the stability of this gene across all the tissue types used in the study [17]. Single dissociation curves were observed for all the flax UGT genes and ETIF5A, confirming amplification specificity of the primers. The ΔC_T_ method [18] was used to express the results relative to the reference gene. A validation experiment was conducted to ensure similar amplification efficiencies of all the genes analyzed.

**Figure 4:**
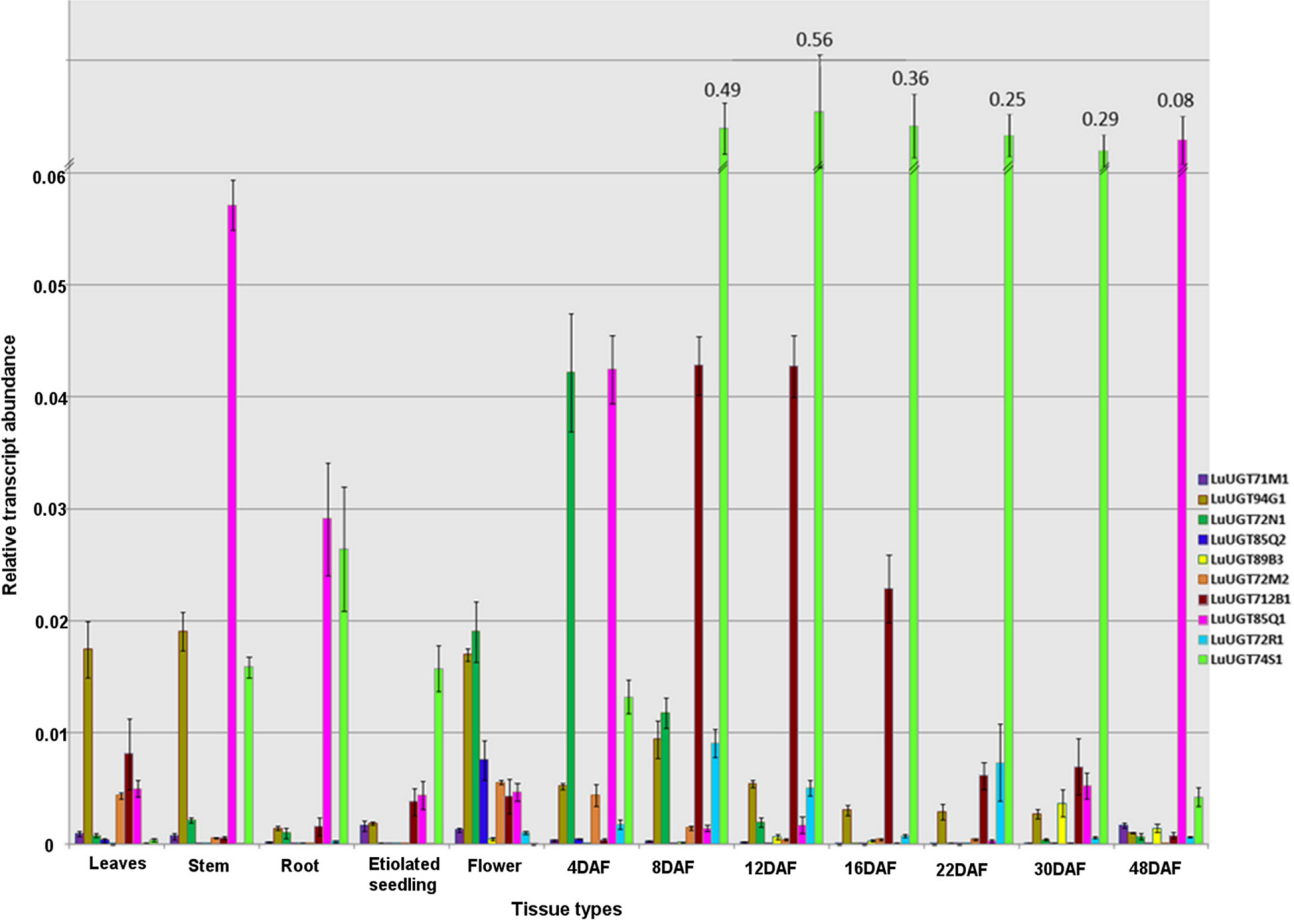
**RT-qPCR expression profile of 10 selected flax UGT genes in 12 different tissue types.** Tissue types analysed for LuUGT expression include; mature leaves (ML), stem (ST), root (RT), etiolated seedling (ES), flower (FL) and seed developmental stages (4, 8, 12, 16, 22, 30, 48 DAF). These graphs show the relative transcript abundance of each gene in comparison with the reference gene, *Linum usitatissimum* ETIF5A (GR508912). Expression values are reported as the average of three biological and two technical replicates. Values correspond to the mean and standard error of biological triplicates.

Relative transcript abundance of 10 flax UGT genes was profiled and is graphically represented in Figure 4. All the selected genes had EST expression evidence and covered six phylogenetic groups. The *LuUGT71M1* transcript was detected in mature leaves, stem, etiolated seedling and 48 DAF; however, the relative expression level compared to other UGT genes was very low. *LuUGT94G1* expressed constitutively in almost all tissues types; specifically it showed maximum expression in stem. Its expression was also supported by ESTs from stem peel library. *LuUGT72N1* expressed in flower, 4 and 8 DAF with peak at 4 DAF. *LuUGT85Q2* had 54 ESTs mapped from flower EST library and RT-qPCR analysis confirmed its high expression in flower. Expression of *LuUGT89B3* was observed in later stages of seed development *viz.* 30 and 48 DAF and supported by two EST clones identified in torpedo seed coat stage. *LuUGT72M2* expressed in mature leaves, flowers and early seed developmental stages whereas *LuUGT72R1* and *LuUGT712B1* were highly expressed in various seed developmental stages. *LuUGT85Q1* belonged to family 85 which is known to be involved in glycosylation of cyanogenic compounds [19]. The abundance of cyanogenic compounds and higher expression of *LuUGT85Q1* in stem, root and mature seed (i.e. 48 DAF) suggest the putative function as cyanogenic glycosyltransferases [20]. *LuUGT74S1* expressed highly in developmental seed stages and peaked at 12 DAF i.e. torpedo stage of embryo. Flax has a major lignan, secoisolariciresinol diglucoside, which is a phenylpropanoid and accumulates in seed coat [21]. UGTs belonging to the gene family 74 glycosylate phenylpropanoid group of compounds. About 25 ESTs clones from torpedo stage seed coat library were mapped on *LuUGT74S1* gene indicating its putative *in planta* function as secoisolariciresinol glycosyltransferase. Expression profiles of the 10 selected genes analyzed using RT-qPCR, matched well with the digital expression results.

## Discussion

Glycosylation mediated by glycosyltransferase enzymes (GTs) is a critical step in metabolic pathways with diverse roles in cellular processes and homeostasis [22]. Recent studies involving functional characterization of plant GTs suggest their important roles in growth, development and interaction with the environment [9]. The activities of many GTs from a variety of plants and biological roles of their products have been known for a long time [23]. However, the methods for identification of UGTs based on biochemical and classical genetic approaches are slow and difficult [15]. Recent developments in plant genomics stimulated the use of strategies such as differential display methods and/or homology-based screening of cDNA libraries for identification and isolation of novel UGT genes [24-26], although the roles of many UGTs still remain uncertain. Availability of whole genome sequence of many plants enabled a thorough and detailed analysis of multigene families. For example, in Arabidopsis, genome-wide search using PSPG motif identified 120 putative UGT genes. Similarly, a whole genome survey of six plant species resulted in identification of 56 (*Carica papaya*) to 242 (*Glycine max*) UGTs [27].

The recently published draft genome sequence and the extensive tissue specific EST library collections of flax provided an opportunity to investigate the diversity in flax UGT multigene family in a greater detail. We identified 137 flax UGTs, which is more than that identified in Arabidopsis but less than that discovered in rice, grapevine and Medicago [27]. All the identified UGTs contain two major domains, a conserved C-terminal domain and a variable N-terminal domain, although the overall sequence diversity was high among the genes.

### Flax UGT family resembles the phylogenetic group structure of Arabidopsis UGTs

A phylogenetic tree provides a framework to compare the properties of gene family members and to identify similarities and differences among them [28]. In the present study, the flax genome revealed 22 UGT families including four new families (94, 97, 709 and 712), not reported in Arabidopsis. However, phylogenetic analysis of flax UGTs clustered them in 14 groups (A-N) as reported in Arabidopsis [7,12] and interestingly, the four new flax UGT families did not form any additional groups. Moreover, all the six sequences of the UGT94 family clustered with the *Sesamum indicum* UGT94D1 sequence (BAF99027 [29]), and UGT94B1 (AB190262 [30]) are the only UGT94 family sequence reported till now. A phylogenetic tree constructed by Bowles *et al.*[31] using 22 UGT sequences reported from other plant species along with the Arabidopsis UGT sequences, mostly resulted in 14 groups, while an additional group of cytokinin GTs was identified containing the *Phaseolus vulgaris* and *Zea mays* UGT sequences [31,32]. Based on the phylogenetic analysis of Arabidopsis UGTs, it has been shown that it might be possible to correlate, to a large extent, the regiospecificity of glycosylation to the phylogenetic groups [33]. The exception to this might be due to regioswitching events taking place during evolution. In some cases, phylogenetically closely related UGTs show distinct regiospecific differences towards a common acceptor. For example, *A. thaliana* UGTs, *AtUGT74F1* and *AtUGT74F2*, share ~82% amino acid sequence identity, and while *AtUGT74F1* glucosylates the phenolic hydroxyl group of 2-hydroxy benzoic acid, *AtUGT74F2* glucosylates both the carboxyl and hydroxyl groups of 2-hydroxy benzoic acid [34]. On the contrary, in some cases (e.g. UGT85B1), the genes have been shown to exhibit a broad specificity toward acceptors *in vitro*; however, a member of this group (UGT85Q1) in *Sorghum bicolor* specifically catalyzes the conversion of *p*-hydroxymandelonitrile into dhurrin *in vivo*[35]. This analysis, along with amino acid sequence similarity of UGT families within a group, might be useful for predicting substrates [31,36]. For example, Osmani *et al.*[37] reported that the group G members glycosylate terpenoids; while the members of groups D, E and L glycosylate flavaonoids, tepenoids and benzoates.

However, a study of several *Medicago truncatula* UGTs highlighted the difficulties in assigning substrate specificity based on phylogeny. Biochemical and phylogenetic studies of *MtUGT78G1* and *MtUGT85H2* showed that substrate specificity could not be predicted by their clustering with biochemically characterized UGTs belonging to the same family [38]. Although, few genomes such as rice, poplar, grapevine and Medicago have been screened and annotated for GT genes, they have not been assigned to GT groups and families so far. Apart from the model plant Arabidopsis [12], this is the first attempt to classify GT genes into groups and families from a crop plant flax, as per the standardized system recommended by the UGT Nomenclature Committee [6]. Thus, the present analysis of flax UGT genes might help to narrow down the substrate choice of a specific gene.

### Detection of orthologs and functional divergence of unique flax UGTs

Detection of orthologs is critically important for accurate functional annotation and has been widely used to facilitate the studies on comparative and evolutionary genomics [39]. Several methods such as the BlastP [40], inparanoid [41] and reciprocal smallest distance [42] have been reported to detect orthologs. In the present study, we used BlastP to identify the orthologs for flax UGTs from four sequenced dicots (*Ricinus communis, Populus trichocarpaVitis vinifera* and *Arabidopsis thaliana*). Of the 137 flax UGTs, 130 UGTs had orthologs from the four dicots and seven flax-diverged UGTs were detected. Based on the microarray and EST data, 95 of these 130 orthologs (73%) showed expression evidence; while, five of the seven flax diverged UGTs revealed expression evidence, suggesting their functional divergence. Thus, the flax diverged UGTs, with significantly different primary sequences than those of other surveyed dicots, might have evolved independently since the last common ancestor between flax and these dicots. As the number of flax diverged UGTs identified in our analysis is small, other methods such as inparanoid search need to be conducted to identify more flax diverged UGTs that the present analysis might have missed. However, we could not perform this analysis, as the flax scaffold sequences are not yet publicly available for conducting the inparanoid search.

### Intron mapping to understand the evolution of UGT family

To understand the evolution of a gene family within phylogenetic groups, introns, more specifically their position, phase, loss and gain, can serve as an important tool [43]. Therefore, we conducted intron mapping in the 137 flax UGTs among which 40.14% sequences were intron less. This percentage is less than that observed in Arabidopsis, wherein >50% genes were intron less [12]. In flax UGTs, a total of seven intron positions were identified with the number of introns per family in the range of one to four. Most families showed the presence of conserved introns 3 (53.65%) and 4 (32.92%), which could probably be considered as the oldest among the seven introns identified. Intron 3 was present in almost all members of the groups F-J and N; while intron 4 was dominant in groups L and K. Interestingly, in these groups wherever intron 3 was present, intron 4 was absent and *vice versa* except in case of *LuUGT709E3*, where both the introns were present; while in case of *LuUGT87J2*, both were absent. In other groups, the introns 3 and 4 were absent in some members of groups A, D, M and E. This suggests that either of these introns was gained prior to diversification of flax UGTs. This is also supported by the observation that most of the conserved introns were in the same phase.

It is a commonly held view that the majority of conserved introns are ancient elements and their phases usually remain unchanged [44]. In fact, it has been further suggested that the intron sliding or shifts of intron-exon boundary over a few nucleotides causing change of intron phase are rare events and introns retain their phase for a long evolutionary time [45]. Furthermore, the introns other than the conserved introns were found only within a single restricted group of closely related sequences or in only a single gene, suggesting a general pattern of intron gain during evolution of the flax UGT gene family. A clear case of loss of a conserved intron and gain of intron 5 was seen in the subfamily of closely related genes *LuUGTB17LuUGTB19* from group A. Similarly, in case of *LuUGT73B12* and *LuUGT73B13*, loss of conserved introns and gain of intron 2 was also observed. Thus, analysis of the evolution of the flax UGT multigene family provides evidence for both intron gain and loss and thereby strongly supports the “intron-late” theory of intron evolution [46].

### Expressed flax UGTs: identified by digital expression analysis and supported by RT-qPCR

Functional divergence among duplicated genes is one of the most important sources of evolutionary innovation in complex organisms. Interestingly, among the 22 duplicated genes, five pairs of genes *LuUGT94G3* and *LuUGT94G4LuUGT73B12* and *LuUGT73B13LuUGT712B1* and *LuUGT712B5LuUGT86A8* and *LuUGT86A9* and *LuUGT74S5* and *LuUGT74S6*, showed evidence of differential expression. For example, *LuUGT74S5* showed seed coat specific expression, while its duplicated counterpart, *LuUGT74S6*, remained unexpressed. Evidence for differential expression was also provided by the duplicated gene pair *LuUGT86A8* and *LuUGT86A9*. This suggests that after duplication, the genes acquired either differential or tissue specific expression patterns. In an earlier study, Haberer *et al.*[47] estimated that about two thirds of duplicate gene pairs had divergent expression in Arabidopsis.

To predict and understand the roles of these UGT genes in various tissue types, gene expression pattern analysis is very helpful to infer which gene family members are expected to perform distinct or similar roles. With this aim, we performed expression analysis of flax UGTs using EST libraries, microarray data and RT-qPCR. About 62% flax UGTs showed expression evidence based on the EST data and one or more ESTs were detected per tissue type, providing strong evidence that most of the flax UGT genes were expressed in varied tissue types. The expression patterns analysed using RT-qPCR very well correlated with the digital expression analysis.

The frequency of ESTs per UGT gene ranged from 1–54 among the UGTs, suggesting varied expression levels. Among the different tissue types, seed and stem tissues showed the highest number of expressed UGTs. It is known that flax seeds and stem contain a large number of secondary metabolites and hence could explain the abundance of UGTs in these tissues [48,49]. However, this could also be due to a large number of EST libraries available for these tissue types (seed: 9 EST libraries, 2,20,724 ESTs and stem: 3 EST libraries, 32,184 ESTs). This study also identified two genes, *LuUGT85Q2* and *LuUGT74S1*, belonging to groups G and L respectively, which showed high expression in flower and seed coat from the torpedo stage. The members of these groups are predicted to glycosylate terpenoids, flavanoids and benzoates classes [37]; and hence, they can be considered as potential targets for screening against these predicted classes to identify their substrates.

Compared to the sequence based expression analysis method, microarray provides a high-throughput tool for simultaneous analysis of expression at the whole transcriptome level. As per the microarray data, 44% flax UGTs showed expression evidence in various tissue types (Figure 3). Three genes from seed stage and one gene from leaf showed high expression, suggesting possible involvement of these genes in seed and leaf secondary metabolite glycosylation. Microarray data from two contrasting flax varieties, Drakkar and Belinka were also analyzed. Drakkar produces better quality fibres than Belinka, and is more resistant to the fungal pathogen *Fusarium*[14]. However, we could not detect any UGT having variety specific expression pattern. Although, plant UGTs have been reported to be involved in defence mechanism [50], the available microarray data were not generated by exposing the varieties to any pathogen. The difference in expression of the UGTs between the EST and microarray datasets might have resulted from the differences in the number of tissue types, size of each dataset and varieties used for data generation. The EST dataset was larger compared to the microarray dataset, therefore we might have obtained expression evidence for more genes using the EST dataset. Moreover, the long sequence reads of ESTs provide fairly unambiguous evidence of gene expression, compared with the hybridization based microarray data and hence EST profiling could be considered as a more reliable method for transcriptomic analysis as also suggested by Geisler-Lee *et al*. [13] and Moreau *et al.*[51].

Regarding the 37 unexpressed flax UGTs, it is possible that some or most of these genes may express at very low levels in particular tissue type or express only under specific conditions such as biotic or abiotic stresses. Hence, they might have not been represented in the EST and microarray data as the data were generated from unchallenged libraries. Even in the large Arabidopsis EST collection gathered over several years, only 64.5% of the genes had corresponding ESTs [52]. Absence of an EST for a corresponding gene implies that it is either inactive or expressed at undetectable level in the tissues sampled or that it is a non-functional gene *per se*.

## Conclusions

We identified a large number of UGT genes in the *Linum usitatissimum* genome. These genes were clustered into 14 distinct evolutionary groups based on the phylogenetic analysis. Two new UGT family members not observed in Arabidopsis were identified in the flax genome. Most of the identified genes were expressed in various tissue types and seven of them were flax diverged. Results of the digital expression analysis were confirmed by RT-qPCR. Two conserved introns were observed, indicating evolution of flax UGTs from two lineages. The phylogenetic tree can be useful for understanding the structure-function relatedness of the UGT family members and might further facilitate their functional analysis.

## Methods

### Probing the flax genome for UGT genes

The presently available draft genome sequence of flax (http://linum.ca) represents 85% genome coverage, which is derived from the low-copy fraction of the genome. This coverage is consistent with the length of the entire low-copy fraction previously estimated by reassociation kinetics [53]. We used the predicted protein database available at http://linum.ca to identify flax UGT genes. The 44 amino acid conserved sequence of the PSPG box that characterizes plant UGTs was used as a query against the 47,912 predicted flax gene models. The resulting scaffolds were analyzed to identify the genes, ORFs, intron positions and sizes using the GBrowse tool available on the same website.

### PCR amplification, cloning and sequencing

Genomic DNA from a flax variety, NL260, was extracted using CTAB method. Total RNA from developing seeds was extracted using Spectrum Plant Total RNA kit (Sigma-Aldrich, USA) and treated with DNaseI (Promega, USA), followed by first strand cDNA synthesis using AMV Reverse Transcriptase (Promega, USA). To confirm the reading frames, primers were designed to amplify full length genes including the start and stop codons (Additional file 8). For intron-less genes, 50 ng genomic DNA, and for intron containing genes, 1.5 μl pooled cDNA from developing seeds was used as template for PCR amplification using AccuPrime^™^*Pfx* DNA Polymerase (Invitrogen, USA). PCR was performed using the annealing temperatures mentioned in Additional file 8. The PCR amplicons were analyzed on 1.0% agarose gels and eluted using GenElute gel extraction kit (Sigma-Aldrich, USA) followed by cloning into pGEM-T Easy vector (Promega, USA). Plasmid DNA was isolated using GenElute plasmid extraction kit (Sigma-Aldrich, USA) and sequenced using MegaBACE 500 (GE Healthcare, UK) DNA analysis system.

### Sequence alignment and phylogenetic analysis

The predicted amino acid sequences of the UGT genes were initially aligned using ClustalW with default gap penalties [54]. These alignments were visually inspected for indels and to minimize insertion/deletion events in unalignable regions. Trees were constructed from 409 alignable amino acid positions (60.41%) for all the sequences. Distance as well as Parsimony analyses were performed using MEGA5 [55]. Only the regions of unambiguous alignments were used in the phylogenetic analyses with Dayhoff substitution matrix (PAM250) and trees were constructed by neighbour-joining algorithm [56] with bootstrapping (1000 replicates). Eighteen Arabidopsis UGT sequences, one from each UGT family and one sesame sequence (UGT94D1) were also included in the analyses (Additional file 9).

### Intron mapping and organization

A flax UGT intron map was constructed by determining the intron splice sites, phases and positions. The introns were serially numbered relative to their positions in the amino acid sequence produced by aligning all the flax UGTs. Intron phases were determined as follows: introns positioned between two codons as phase 0, introns positioned after the first base in the codon as phase 1, and introns positioned after the second base in the codon as phase 2.

### Detection of orthologs of flax UGTs in four sequenced dicots

Blast2Go [57] was used to search the orthologs for flax UGTs in four sequenced dicots, *Ricinus communis* (*Euphorbiaceae*), *Populus trichocarpa* (*Salicaceae*), *Vitis vinifera* (*Vitaceae*) and *Arabidopsis thaliana* (*Brassicaceae*), using default parameters except for E value cut off of < e^−100^. These four dicots were selected based on the genome homologies with flax as reported by Ragupathy et al. [58].

### Digital expression analysis

The putative UGT coding sequences were BLAST searched against the *Linum usitatissimum* NCBI-EST dataset (dated: June, 2011; 2,86,895 sequences; http://www.ncbi.nlm.nih.gov/nucest?term=Linum%20usitasimum) to identify transcriptional evidence for individual UGT genes and to estimate the number of ESTs expressed per tissue type and gene model. These tissue types include flower (FL), globular embryo (GE), heart embryo (HE), torpedo embryo (TE), bent embryo (BE), mature embryo (ME), seed coat at globular stage (GC), seed coat at torpedo stage (TC), pooled endosperm (EN), etiolated seedling (ES), stem (ST), leaf (LE), peeled stem (PS) [59], 12 days DAF bolls and outer fibrous stem tissue. Additionally, microarray expression data for 48,021 flax unigenes (http://www.ncbi.nlm.nih.gov/geo/query/acc.cgi?acc=GSE21868) were also used. RMA - normalized, averaged gene-level signal intensity (log2) values for the unigenes exhibiting specified sequence similarity were used from all the biological as well as technical replicates and averaged further. A heat map for digital expression analysis was constructed with these values using TIGR MultiExperiment Viewer (MeV, http://www.tm4.org/mev.html).

### Reverse transcription quantitative real time PCR

Total RNA from mature leaves (ML), stem (ST), root (RT), etiolated seedling (ES), flower (FL) and seed developmental stages (4, 8, 12, 16, 22, 30, 48 DAF) of flax variety NL260 was isolated as described earlier. DNaseI treated total RNA was reverse transcribed using oligo(dT) primer and MultiScribe^™^ reverse transcriptase (Applied Biosystems, USA). Gene specific primers for 10 glycosyltransferase genes (Additional file 8) were designed using Primer3 [60]. PCR conditions were optimized for annealing temperature and primer concentration. Primers used for real-time PCR are listed in Additional file 8. Real-time PCR was carried out in 7900HT Fast real-time PCR system (Applied Biosystems, USA) using FastStart universal SYBR green master mix (Roche, USA). Each 10 μL real-time PCR cocktail contained 0.125-0.4 μM concentrations of both forward and reverse gene-specific primers (Additional file 8), 4 μL of 1:16 diluted first strand cDNA, 1× SYBR green master mix and sterile milliQ water to make up the reaction volume. Real-time PCR amplification reactions were performed with following conditions: 95°C denaturation for 10 min, followed by 40 cycles of 95°C for 3 s, with primer annealing and extension at 60°C for 30 s. Following amplification, a melting dissociation curve was generated using a 62–95°C ramp with 0.4°C increment per cycle in order to monitor the specificity of each primer pair. Eukaryotic translation initiation factor 5A (*ETIF5A*) gene from flax was used as a housekeeping or reference gene for all the real-time PCR reactions [17]. Housekeeping gene was selected after confirming the stability of this gene across all the tissue type used in the study. For each biological replicate, two independent technical replications were performed and averaged for further calculations. PCR conditions were optimized such that PCR efficiencies of housekeeping gene and the gene of interest were similar and closer to 2.0. PCR efficiencies were calculated using LinRegPCR [61]. Relative transcript abundance calculations were performed using comparative C_T_ (ΔC_T_) method as described by Schmittgen and Livak [18].

## Competing interests

The authors declare that they have no competing interests.

## Authors’ contributions

VTB performed database searches to obtain the UGT sequences and performed cloning and RT-qPCR. VTB and VCP performed various bioinformatics analyses and drafted the manuscript. SMK and NYK helped in data analysis and improved the study design. VSG designed, coordinated and supervised the study. All authors have participated in writing and revision of the manuscript, and have read and approved the final version of the manuscript.

## Supplementary Material

Additional file 1 Summary of 137 flax UGTs: information of genes and intron positions. Click here for file

Additional file 2 Sequence similarity of the phylogenetic groups and families of 137 flax UGTs. Click here for file

Additional file 3 Orthologues of flax UGTs identified from four sequenced dicots. Click here for file

Additional file 4 Information about duplicated genes identified and their differential expression patterns. Click here for file

Additional file 5 Figure S1. Complete amino acid alignment of 137 Flax, 19 Arabidopsis and 1 Sesame UGTs. Click here for file

Additional file 6 Distribution of intron sizes in the flax UGTs. Click here for file

Additional file 7 Summary of digital expression analysis with EST and microarray data. Click here for file

Additional file 8 Information about primers used to clone and sequence full length UGTs and RT-qPCR. Click here for file

Additional file 9 Accession numbers of proteins sequences encoded by genes included in the phylogenetic analysis. Click here for file
