# Supplementary material for: Phylogenomic analysis of UDP glycosyltransferase 1 multigene family in Linum usitatissimum identified genes with varied expression patterns
Source: BMC Genomics. 2012 May 8;13:175. doi: 10.1186/1471-2164-13-175 (PMC3412749; doi:10.1186/1471-2164-13-175)
Supplement: Additional file 2 — Sequence similarity of the phylogenetic groups and families of 137 flax UGTs. [file 1471-2164-13-175-S2.doc]

**Additional file 2: (a)** Sequence similarity of the phylogenetic groups of 137 flax UGTs

| **Sr. No.** | **Group** | **Included families** | **Number of sequences in group** | **% Similarity of conserved 409 AA positions** | | | **% Similarity of full length protein sequences** | | |
| --- | --- | --- | --- | --- | --- | --- | --- | --- | --- |
|  |  |  |  | **MIN** | **MAX** | **AVERAGE** | **MIN** | **MAX** | **AVERAGE** |
| 1 | A | 79, 91, 94 | 16 | 39.9 | 96.3 | 56.3 | 40.2 | 95.4 | 54.6 |
| 2 | B | 89 | 5 | 63.9 | 96.1 | 73.1 | 38.8 | 95.6 | 62.2 |
| 3 | C | 90, 97 | 6 | 61.9 | 94.8 | 72.4 | 57.7 | 93.5 | 68.5 |
| 4 | D | 73 | 21 | 59.8 | 97.9 | 68.3 | 56.0 | 97.2 | 64.4 |
| 5 | E | 71, 72, 88 | 22 | 50.5 | 98.4 | 62.6 | 44.6 | 97.0 | 58.3 |
| 6 | F | 78 | 1 | - | - | - | - | - | - |
| 7 | G | 85, 709 | 19 | 52.5 | 91.9 | 68.6 | 49.8 | 90.7 | 64.9 |
| 8 | H | 76 | 6 | 62.8 | 98.1 | 71.4 | 60.0 | 97.8 | 68.3 |
| 9 | I | 712 | 9 | 64.4 | 90.3 | 76.1 | 60.1 | 89.1 | 73.2 |
| 10 | J | 87 | 4 | 62.0 | 77.0 | 69.2 | 58.1 | 73.4 | 64.9 |
| 11 | K | 86 | 5 | 87.7 | 92.5 | 90.4 | 85.4 | 89.3 | 87.8 |
| 12 | L | 74, 75, 84 | 19 | 48.9 | 96.4 | 61.5 | 45.6 | 94.4 | 58.3 |
| 13 | M | 92 | 3 | 65.5 | 95.6 | 75.9 | 60.5 | 94.9 | 72.0 |
| 14 | N | 82 | 1 | - | - | - | - | - | - |
| **All UGTs** | | | **137** | **37.4** | **98.4** | **52.6** | **36.2** | **97.8** | **49.8** |

**Additional file 2: (b)** Sequence similarity of the families of 137 flax UGTs

| **Sr. No.** | **Family** | **Phylogenetic**  **Group** | **Number of sequences in family** | **% Similarity of conserved 409** **AA positions** | | | **% Similarity of full length protein sequences** | | |
| --- | --- | --- | --- | --- | --- | --- | --- | --- | --- |
|  |  |  |  | **MIN** | **MAX** | **AVERAGE** | **MIN** | **MAX** | **AVERAGE** |
| 1 | 79 | A | 6 | 47.5 | 96.3 | 64.2 | 49.6 | 95.4 | 63.8 |
| 2 | 91 | A | 4 | 62.9 | 96.2 | 74.2 | 59.5 | 92.7 | 71.3 |
| 3 | 94 | A | 6 | 51.3 | 93.6 | 70.1 | 49.2 | 90.3 | 66.2 |
| 4 | 89 | B | 5 | 63.9 | 96.1 | 73.1 | 60.5 | 95.6 | 70.4 |
| 5 | 90 | C | 3 | 75.5 | 77.7 | 76.5 | 67.9 | 73.0 | 70.3 |
| 6 | 97 | C | 3 | 89.9 | 94.8 | 92.2 | 86.8 | 93.5 | 89.7 |
| 7 | 73 | D | 21 | 59.8 | 97.9 | 68.3 | 56.0 | 97.2 | 64.4 |
| 8 | 71 | E | 7 | 61.2 | 93.2 | 69.6 | 56.8 | 93.6 | 66.5 |
| 9 | 72 | E | 13 | 59.8 | 98.4 | 69.2 | 56.3 | 97.0 | 65.6 |
| 10 | 88 | E | 2 | 73.2 | 73.2 | 73.2 | 69.0 | 69.0 | 69.0 |
| 11 | 78 | F | 1 | - | - | - | - | - | - |
| 12 | 85 | G | 15 | 63.4 | 91.1 | 74.1 | 60.1 | 89.8 | 70.2 |
| 13 | 709 | G | 4 | 59.8 | 91.9 | 71.1 | 59.4 | 90.7 | 68.8 |
| 14 | 76 | H | 6 | 62.8 | 98.1 | 71.4 | 60.0 | 97.8 | 68.3 |
| 15 | 712 | I | 9 | 64.4 | 90.3 | 76.5 | 60.1 | 89.1 | 73.2 |
| 16 | 87 | J | 4 | 62.0 | 77.0 | 69.2 | 58.1 | 73.4 | 64.9 |
| 17 | 86 | K | 5 | 87.7 | 92.5 | 90.4 | 85.4 | 89.3 | 87.8 |
| 18 | 74 | L | 12 | 57.8 | 90.8 | 66.8 | 55.5 | 91.7 | 64.1 |
| 19 | 75 | L | 4 | 71.4 | 96.4 | 81.3 | 68.9 | 93.7 | 78.3 |
| 20 | 84 | L | 3 | 91.5 | 96.3 | 93.3 | 87.4 | 94.4 | 90.3 |
| 21 | 92 | M | 3 | 65.5 | 95.6 | 75.9 | 60.5 | 94.9 | 72.0 |
| 22 | 82 | N | 1 | - | - | - | - | - | - |
