# Supplementary material for: Phylogenomic analysis of UDP glycosyltransferase 1 multigene family in Linum usitatissimum identified genes with varied expression patterns
Source: BMC Genomics. 2012 May 8;13:175. doi: 10.1186/1471-2164-13-175 (PMC3412749; doi:10.1186/1471-2164-13-175)
Supplement: Additional file 4 — Information about duplicated genes identified and their differential expression patterns. [file 1471-2164-13-175-S4.doc]

**Additional file 4:** Information about duplicated genes identified and their differential expression patterns

| **Sr. No** | **Phylogenetic**  **group** | **Duplicated gene**  **pair names** | **Overall %**  **sequence similarity** | **Intron no.**  **present in**  **duplicated pair** | **Differential expression remark** |
| --- | --- | --- | --- | --- | --- |
| 1 | A | LuUGT79B18/LuUGT79B19 | 95.4 | 5/5 | Same expression Pattern |
| 2 | A | LuUGT91J1/LuUGT91J2 | 92.7 | 3/3 | Same expression Pattern |
| 3 | A | LuUGT94G3/LuUGT94G4 | 90.3 | 0/0 | G3 constitutively expressed in all tissue types and G4 remain unexpressed. |
| 4 | B | LuUGT89B3/LuUGT89B4 | 95.6 | 0/0 | Same expression Pattern |
| 5 | C | LuUGT97B2/LuUGT97B3 | 93.5 | 0/0 | Same expression Pattern |
| 6 | D | LuUGT73Z2/LuUGT73Z3 | 97.2 | 0/0 | Same expression Pattern |
| 7 | D | LuUGT73W1/LuUGT73W2 | 94.2 | 0/0 | Both are unexpressed |
| 8 | D | LuUGT73B12/LuUGT73B13 | 93.3 | 2/2 | B12 expressed in seed and B13 remain unexpressed |
| 9 | D | LuUGT73Z4/LuUGT73Z5 | 96.8 | 0/0 | Both are unexpressed |
| 10 | D | LuUGT73W3/LuUGT73W4 | 95.4 | 0/0 | Both are unexpressed |
| 11 | E | LuUGT71A24/LuUGT71A25 | 93.6 | 0/0 | Same expression Pattern |
| 12 | E | LuUGT72R1/LuUGT72R2 | 92.1 | 4/4 | Same expression Pattern |
| 13 | E | LuUGT72T1/LuUGT72T2 | 97.0 | 0/0 | Both are unexpressed |
| 14 | G | LuUGT709E2/LuUGT709E3 | 90.7 | 3/3&4 | Same expression Pattern |
| 15 | H | LuUGT76N2/LuUGT76N3 | 97.8 | 3/3 | Same expression Pattern |
| 16 | I | LuUGT712B1/LuUGT712B5 | 89.1 | 3/3&5 | B5 expressed in stem and leaves |
| 17 | K | LuUGT86A8/LuUGT86A9 | 89.3 | 4/4 | A9 highly expressed in stem parts and leaves |
| 18 | K | LuUGT86A6/LuUGT86A7 | 89.3 | 4/4 | Same expression Pattern |
| 19 | L | LuUGT74S5/LuUGT74S6 | 91.7 | 4/4 | S5 expressed in seed coat while, S6 remain unexpressed |
| 20 | L | LuUGT75N1/LuUGT75N2 | 93.7 | 0/0 | Same expression Pattern |
| 21 | L | LuUGT84G2/LuUGT4G3 | 94.4 | 0/0 | Same expression Pattern |
| 22 | M | LuUGT92A3/LuUGT92A4 | 94.9 | 0/0 | Same expression Pattern |
