# Supplementary material for: Phylogenomic analysis of UDP glycosyltransferase 1 multigene family in Linum usitatissimum identified genes with varied expression patterns
Source: BMC Genomics. 2012 May 8;13:175. doi: 10.1186/1471-2164-13-175 (PMC3412749; doi:10.1186/1471-2164-13-175)

Additional Figure 1

Complete amino acid alignment of 137 Flax, 19 Arabidopsis and 1 Sesame UGTs

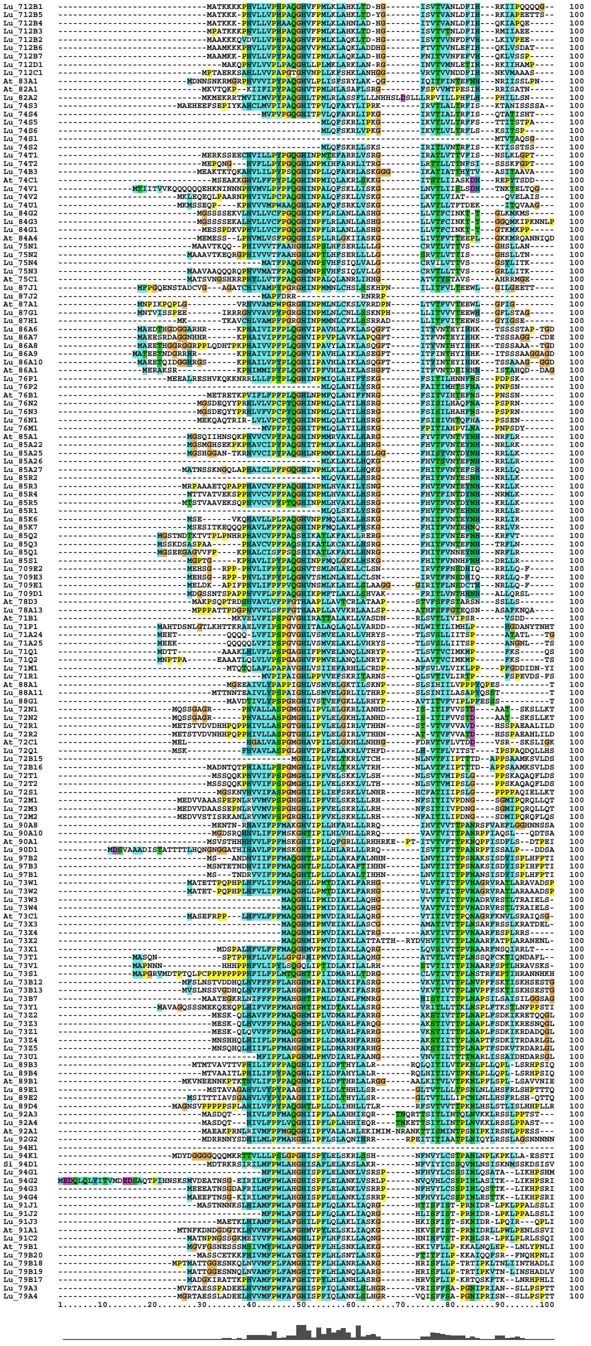

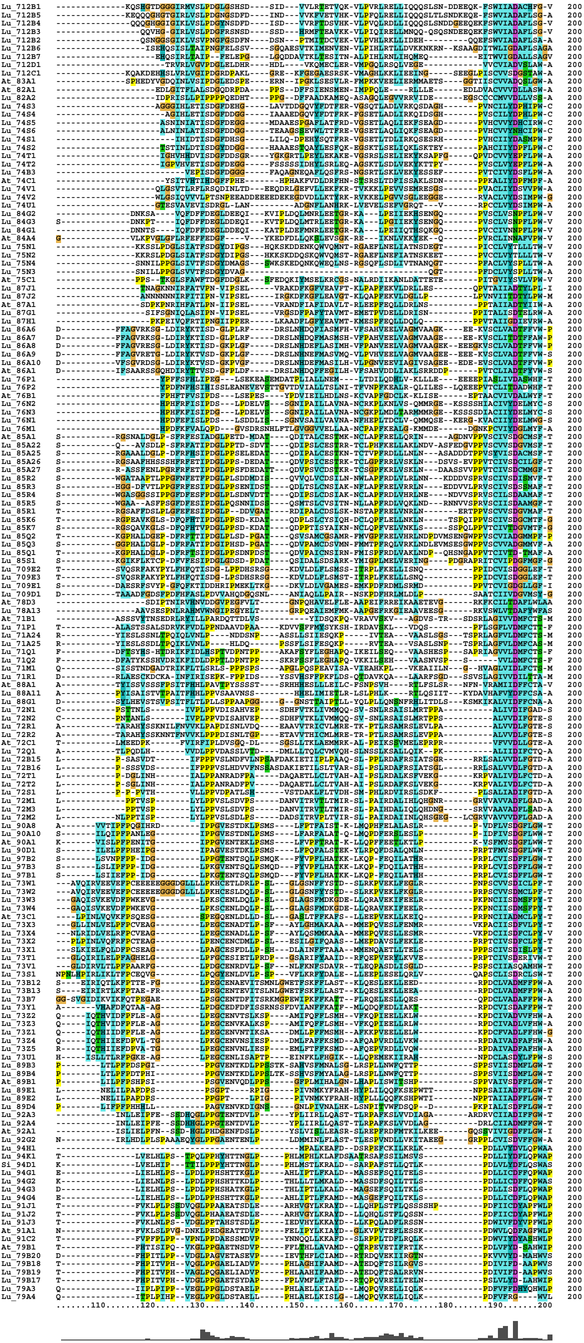

```
Lu_712B1    FIVAREMGIK-TVALWTASQENLALVLRIPQLIETGTIN-ENGF-LVDKELP----ISISEEMVAWKANELPWSAPS--EELQSFYFKNCYSKPSEHCSL   300
Lu_712B5    FVVAKELGIK-TAALWTAAMENFALMLRIPQLIEAGTID-ENGF-STDKELP----ISISEEILAWKANELPWSVQP--EERQTVFFNTSYTHPSKHISL   300
Lu_712B4    FVVAKEMGIK-TVALWTASLENFALMLRIPQLIEAGTID-ENGF-LTDKELP----ISISDEILAWKANELPWSCQS--EEFQSFLFKNFYTQPSKHCFL   300
Lu_712B3    FIVAKEMGIK-TAALWTASLENFALMLHIPQLIEAGTID-ENGF-LIEKELP----VSIYNEMLAWKANELPWSYQP--EELQPFLFKNYYAQPSKHCLL   300
Lu_712B2    FVVAKEKGIR-TTAFWTASMENLASILRIPQLIQDGTID-ENGS-LINEDLP----ISLCREIPSWKANELPWSCQP--DEIQSFMFRRYYVNPAKYFAL   300
Lu_712B6    FQVAKEMGIK-TAAFWTGSAATLALLLRIPQLIQDGILD-ENGT-LINRGMP----ICLSKDIPAWQPDEFPWSCQP--EQFQRFGFKAFSSKPSENSTL   300
Lu_712B7    FQVAKELGIK-TAAFWTASMENLAFLLSIPQLIQDRIID-EKGT-LINSSWP----VCLSKDIPSWQPNELPWSCQP--EEFQRFIFKNYSLKPSQNSAL   300
Lu_712D1    FHEAKAMGTK-TAAFYPASAATLSLLLDIPRLLQLRILD-HDGV-GLTESS-----IGMAKEIPSWEANELPWSHPAYTDELRKLSFQSCCFNVRECSQN   300
Lu_712C1    LEIGREMGIK-CGVVSPVAVINLSLTLHIPKLIQSGILS-PHGLPLKNEAIV----LPNQGELPPWQPNELPWHHPN--PQVQKHLFKQYTLKQLAILPQ   300
At_83A1     IEVAAKFGIR-RTAFCPAAAASMVLGFSIQKLIDDGLID-SDGT--VRVNKT----IQLSPGMPKMETDKFVWVCLK-NKESQKNIFQLMLQN-NNSIES   300
At_82A1     IGVADRCGVP-VAGFWPVMFAAYRLIQAIPELVRTGLVS-QKGCPRQLEKTI------VQPEQPLLSAEDLPWLIGTPKAQKKRFKFWQRTLERTKSLR-   300
Lu_82A2     LDVARRCGVP-VAGFWPASLLSYRLIAAIPQLVASGIIHPHTGIPQHQGLSI----GGLEPNQPPLTSDDLPWLIGSLTARKARFKFWTKTLHRSTNLQ-   300
Lu_74S3     LDVSKRFGLI-GAAFFTQSCAVDAVFYHVHRGLLK-PPVTE-VE--ETVSIP----GLP-----PFEPHDLPSFVHD-GSY---PAFLAALVGQFSNIQN   300
Lu_74S4     LDVSKRFGLI-GVAFLTQSCTVDVVFYHVHHGLLK-PPVTQ-VE--ETTSIP----GPP-----PLDPADLPSFVHD-GSY---PAFLALAVGQFSNIQN   300
Lu_74S5     LHVAKRFGLV-AAAFLTQSCAVDCVYKLVHDGVIKTPPVKE-KD--GALRLE----GLP-----PLTARDLPSFVSDVGSY---PGIRDALVGQFENMED   300
Lu_74S6     LDVAKRFDLL-AAAFLTQSCAVDCVYKLIHEGVVK-PPVKE-ED--GVLRFE----GLP-----PLTAGDLPSLVSDVGSY---GAVLDALVGQYENIKD   300
Lu_74S1     LDVAKRFGIV-GAAFLTQSCAVNAIYYHLREGTIKRPVVSDPAA--GTLVID----GLP-----PLEVSDLPSFIWD-DLH---TEFLAAHLRQFSN-DG   300
Lu_74S2     LDVAKELGLF-AAPFFTQSCAVDAIYYHVYKGSLKLPVTDQ--P--QSLIIP----GLPA----PLEADDMPSFISDYGSY---PAAFDMIISQFSNIHK   300
Lu_74T1     LDVAKEHGLY-AAPFFTQPCAVDYVYYNVWAGSLG-LPVDG-----WPVEIP----GLP-----VMEAADAPSFLVDPVSS---KDFLGLLVNQFSNAER   300
Lu_74T2     LDVAKEHGLY-AASFFTQPCAVDFIYYNIRHGLLK-LPVDT-----WPVRIL----GLP-----ELEPRDMPSFVNAPEAY---PAYFAMVVNQFSNTEK   300
Lu_74B3     LDVAKQYGVY-GAAFFTNSAAVCGIFCRVSRGEVALEMAAK--E--GGLDFP----GLP-----SLGLSDLPSFLRFPESY---PTYLGMKLCQYSNLEE   300
At_74C1     LDIAKDLDLY-VVAYFTQPWLASLVYYHINEGTYDVPVDRHENP--TLASFP----GFP-----LLSQDDLPSFACEKGSY---PLLHEFVVRQFSNLLQ   300
Lu_74V1     LGIAKEMNIL-GAPFFTMPCAVDTIFYNYHQGEIKLRRPMDDNDKKAKIRVE----GIEEV---ELEIQDLPSYLHDDVDV-NTPQSLTLLSDQFSNVAD   300
Lu_74V2     LGIARKLNLA-GAPFFTQPCAVDAIFCSHYEGTLKIP-VGDDRD----VCVE----GMGR----MLDLHDLPCLLYETG---TMPGALDLLSRQFSTVAD   300
Lu_74U1     VGIARELGMV-GAAFFTQPAAVNGVFLEVMEGRIGVP---PEKG--MVTEVE----GWPA----AMEVCDLPSFVSDVLDSPSRRMGLEMMAGQFSTARE   300
Lu_84G2     SDVAVSLDIP-SAILWMQSCACFSSYYHYHN--KLARFPTENEPE-CDVVLP----SMP-----VLKHDEVPSFLHPSTPHPFLATAILGQIAFLG---K   300
Lu_84G3     CDVAVSLDIP-SAILWMQSCACFSSYYHYHN--KLARFPTENDAE-CDVVLP----SMP-----VLKHDEVPSFLHPSTPYPFLATAILGQFAYLD---K   300
Lu_84G1     SDVAASLDIP-SAIFWMQACASFSCYYHYYK--KLARFPTEDDPE-SDVVLP----FMP-----VLKHDDIPTFLLPSTPYPYLATAVFDQFAYLDND-K   300
At_84A4     CDIAEELQIP-SAVLWVQSCACLAAYYYYHH--QLVKFPTETEPE-ITVDVP----FKPL----TLKHDEIPSFLHPSSPLSSIGGTILEQIKRLH---K   300
Lu_75N1     ADVARDNNLP-SVLLWIQPATVFDIYYYLAN-GYEEAFEKCRNPS-FRLELP----GIP-V---SFTNDELPSFASPCNPHPLLRQAMIEQVKVLTRDDG   300
Lu_75N2     ADVARDNTLP-SVLLWIQPATVFDIYYYLTN-GFEEAFEKCKDPS-FELELP----GIP-V---SFTNDELPSFASPSNPHPFLRHAMIEQVKVLTRDNG   300
Lu_75N4     VDVARDHNLP-TTLLWIQPATVMDIYYYLFN-GYGDLFEKCKDPS-FAMDLP----GLHSV---SFTSKDLPSFAIHPNQYPLLINGVKQQMQVLTRDGT   300
Lu_75N3     VDVGRDLDLP-TTLLWIQPATVMDIYYYLFN-GYGELFEKCKDPS-FSMDLR----GLDSV---SFTSNDLPSFAIHPNQYPLLINGVKQQLQVLTRDGT   300
At_75C1     STVAREFHLP-TTLLWIEPATVLDIYYYYFNTSYKHLFD--VEP----IKLP----KLP-----LITTGDLPSFLQPSKALPSALVTLREHIEALETE-S   300
Lu_87J1     CEIGVRRGIP-VASLWTMSATVFSVLLHFDLVRQHRHFPIDDFSGIEEEKVD----YIPG----LSPTRIVDLQP-FLISRIG--VMDKALQSVSLVSK-   300
Lu_87J2     CDVGSSRGIP-VASLWTMPATVFSVFLHFDLLRQHRHFPISDLPEIEEEKVD----YIPG----LPPTPILDIEP-VITGLIRQEVLHIALESVSVASKR   300
At_87A1     VRVGTKRNIP-VASFWTTSATILSLFINSDLLASHGHFPIEPSESKLDEIVD----YIPG----LSPTRLSDLQ---ILHGYSHQVFNIFKKSFGELYK-   300
Lu_87G1     IRIGNRRKIP-VATLCTVPAGVFSVFHRFAAIQDFKLRDSVLDEEEMG-YRP----SNHE----ISPSEIADLK--AIFRGDDRRIMGLTLECISWVPK-   300
Lu_87H1     IGVGTRRNIP-VAAFWTMSASFFSMLYHLDVS--AKAHNSSP-EDLID-CGD----LLPG----ISTSRVSELK--KLFQKNDLRVLQLAIECVSKVTK-   300
Lu_86A6     SKVAKKFGLV-FVSIWTQPALVFTLYHHVHLLRRNCHFGCQDRREDAIEYIP----GVKR----IEPKDMPSILQ----EVDEN--VEKTAFVAFRDVRY   300
Lu_86A7     SKVVKKFGLV-FVSIWTQPALVFTLYHHVHLLRQNGHYGCQDRREDSIDYIP----GVKK----IEPKDLPSILQ----EIDETSLFIQATFHVLQDVKS   300
Lu_86A8     SKVAKKFGLV-YVSVWTQPALVFTLYHHVHLLRQNGHFGCRDRRKDTIDYVP----GVKR----IEPKDTPSPLQ----EDDETTIVHQTTLGAFHDVRS   300
Lu_86A9     SKVAKKFGLV-YVSVWTEPALVFTLYHHVHLLRQNGHFGCQGRRDDPIDYIP----GVKI----IEPKDTPSSLQG---DDDETVIDHQVVFGAIQDAKS   300
Lu_86A10    SKVAKKFGLV-YVSVWTEPILVYTLYHHVHLLRQNGHYGCKDRRKDAIDYIP----GVKR----IEPKDTMSFLQ----EADENTIIHQIIFPAFQDARG   300
At_86A1     SMICDKHNLV-NVSFWTEPALVLNLYYHMDLLISNGHFKSLDNRKDVIDYVP----GVKA----IEPKDLMSYLQVSDKDVDTNTVVYRILFKAFKDVKR   300
Lu_76P1     QEVADDLKLS-RFVLRTSNACSFLVYNAFPLLLEKGYMPVTDSRLEELVPEL----------PPLRVKDLPDIKMKKPDDFYNLVAGMIRTVNASS---   300
Lu_76P2     QEVADSLRLS-RIVLRTSNVSSFLAYEPLPLFYEKGYLPVQACRADEEIPEF----------PPLKAKDLPQVETQRKDDMLHLVDSMMRTIKASA---   300
At_76B1     HDLTEKFNFP-RIVLRTVNLSAFVAFSKFHVLREKGYLSLQETKADSPVPEL----------PYLRMKDLPWFQTEDPRSGDKLQIGVMKSLKSSS---   300
Lu_76N2     EAVAKSLGLP-SIMLRTNTVSTFIARDHVLKLIDQGRVPLQDSILDQPVPTH----------YPLRYKDLPTSIFKPVTNFIEIVN-NLREVRSSS---   300
Lu_76N3     EAVAKSLGLP-SVMLRTNTVSTFIARDHVLKLIDQGRVPLQDSILDQPVPKH----------YPLRYKDLPISIFKPVTNFIEIVN-NLREVRSSS---   300
Lu_76N1     EAVANSLGLS-SIMLRTNTVSAQLGRNLVLQLMRDGLVPLQDSLLQEPVPDH----------YPLRYKDLPVSHFKPAQNFEEIVT-KISDVRSSS---   300
Lu_76M1     EGVGKEMGIP-SLVLRTSCAANLLTYHVFPQLREKGHLPEQYSTSSEPVPGL----------PNLRYKDLPSYTTNWPIEAQLDFFATVRQTRSAT---   300
At_85A1     LDVAEELGVP-EVLFWTTSGCAFLAYLHFYLFIEKGLCPLKDESYLTKEYLEDTVIDFIPTMKN-VKLKDIPS-FIRTTNPDDVMISFALRETERAK--R   300
Lu_85A22    LDAAEELGVP-EVLFWTTSACGFLGYMHYRDLIDRGIVPLKDESYLTNGYLD-TVIDWIPAMSN-IKLKDLPT-FLRTTNPDEFMVEFVLGETERSRSPK   300
Lu_85A25    LDAAEELGIP-EVVFWTPSACGVLGYANYRRLAEEGLVPLKDEKDLTNGYLN-TPVDWIPAMQG-IQLKNFPN-FIRTTNANDTMFNFLRREIDRTS--R   300
Lu_85A26    VQVAKELGIP-NVMFWTASACGFLGFLNYCKLLEKGIFPLKDASMITNGYLD-TTIDWIPGMEG-IPLKYMPT-FLRTTDPNDVMFNFAMGQVENSR--N   300
Lu_85A27    MQVAKELGIP-NVMLSTASACGFIGYLNYRKLLQKGIVPLKDASYLTNGYLE-TRIDWIPGMEG-IPLKYMPS-FVRTTDPEEFMFNFAMEEVENTQ--N   300
Lu_85R2     LDVAKELGIP-DALFSAMNACATLAYLSSHRLLERGLVPLKDSSYITNGYLE-TIVDCIPGLNKNVRLKDLPTPVVRITDRNDTVFNFALKKIKRIS--E   300
Lu_85R3     LDVSKELGIP-NALFSTPSACASLVYLNYNRLVETGLVPLKDSSYLTNGYLE-TIIDCIPGLNKNIRLKDLPT-FVRITDPNDIIFNFCLKELARIH--K   300
Lu_85R4     LDVAKELGVP-DALFLTPSACANLGFLSYHVLVKRGLVPLKNSSYLTNGYLD-TVVD-IPGLNKNMCLKHLPT-FVRTTDPNDVVFNFCVNELARIP--E   300
Lu_85R5     LDVARELGIP-DALFLCPSACANLPLLSYPVLVERGLVPLKDSSYLTNGYLD-TVVDCILGLNKNMRLKDLPT-FMRTTNPNDVVFNFCIDQLARIP--E   300
Lu_85R1     LEVADELGIP-DVLFWTPSACGVLAYVNYQLLAQRGLVPLKDSSDLKSGYLD-TTVDFITGLNKNIRLKDLPS-FIRTTDTNNIMFNFLSKEASKIR--K   300
Lu_85K6     IKAAELLGIT-QATFWTASACSFMGSLQFEQLVRRGISPLKEAN-LTDGTLD-LHLDWIPGMSN-IRLKDLPS-FATTTDAEDVMFKFAEIEIENCL--K   300
Lu_85K7     IQAAELLGIP-HASFWTASACGMMGYLQFEELITRGIFPLKDVN-FTDGTLE-RRLDWVTGMSD-IRLRDLPS-FATSTDAKDVMFHILKSEAASCL--K   300
Lu_85Q2     LVVAKEIGVP-SLSYWTFPACAFMGFKQYRSLYDQGITPFKDESFRTNGDLE--TPIQVPGMKN-MRLRDLPD-FFQTTDPNEPLLQNLITGTDAVD--I   300
Lu_85Q3     LEVAREIGVP-SLSYWTFAACGFMGFKQYRPLVDQGVTPFKDDSYLTNGFLD--KAVEVPGMKN-MRYRDLPT-FIQTTDPKEPIFHNLMLGAEAVP--I   300
Lu_85Q1     VDVAREFGIP-SVAYWSFAACGFMGFKQFKPLLDQGITPFKDDSYLTNGYLE--TPFEVPGMKD-IRLRDLPS-FFRTTDPDDQVFYCLMEVAEAAH--R   300
Lu_85S1     LVAAERLGVP-GVPFWTASACGFMAYLHIGQLIEKGLIPHKSESYESDGSLD-TEVGWIPGMSH-ARLRDLPC-ATRTTNPEAILLNCLRDEVQADL--R   300
Lu_709E2    VEVADEVGIP-LVYFRTIGASCFWVYFCIPDIIEAGELPIRVE-EEEDMERV---ITKVPGAEGFLRCRDLPS-LCRVGDLSDPFLQAIVSTTRKSP--K   300
Lu_709E3    VEVADEVGIP-LVYFRTIGASCFWIYFCIPDMIEAGELPIRVEVEEEDMERV---ITKVPGAEGVLRCRDLPS-LCRVGDLSDPFLQAIVLTTRKSP--K   300
Lu_709E1    REVSMELGIP-VIRFRTISPCCFWVNYCLPDLIEAGELPI------QDMDRK---ISKVPGMESFLRSRDLPG-MCRVSGLDDPTLVMLINATRESP--P   300
Lu_709D1    IEVAEEIGIP-AITFRTFSAVGLWVYFNLDKLTEDGSIPIPGN---ADMDEL---ITSIPGLEGVLRLRDLPS-MCRPG-PSSQVLKFFIDETKSMK--R   300
At_78D3     ETAAAEMKAS-WVAYYGGGATSLTAHLYTDAIRENVG--VKEVGERMEETIG---------FISGMEKIRVKDTQEGVVFGNLDSVFSKTLHQMGLALPR   300
Lu_78A13    -----EMGIP-WIAFWTAGPNSLSAHLHTDPLREALGSVPDAVVGREEETLKG--------IVAGMSKLRFRDLPEGVVKGNLQSIFSTMLHNMATHLPK   300
At_71B1     IDIADEFNLS-AYIFYTSNASYLGLQFHVQSLYDEKELDVSEFKDT-EMKFDVP----------TLTQPFPAKCLPSVMLN--KKWFPYVLGRARSFRA-   300
Lu_71P1     IDVAVEFGLP-SYVFYTSGAGCLNLTLYFQNLRDAQNVPVSDFNNP-VADWKIE----------GFANSIPGKVLPRPVLN--PYQCDGFLNFVQNYRN-   300
Lu_71A24    LEVADEFNVP-SYIFFTSGAAFLGFMLRIQSLHDDEGFD---VTESEEAELVIP----------SYSNPVPRKVFPSTVLK--KDWAAVLYRLARDFRK-   300
Lu_71A25    LEVADEFSVP-SYIFFTSGAAFLGFMFRIQSLHDEEGYD---VTESEETELVIP----------SYSNPVPRKVFPSTVRK--KEWVDVLYKLARDFRK-   300
Lu_71Q1     MADAKDLGVP-SYVFYTCGATFLGMMFQLQALYDEGLYDPVNMKDSETELLEIP----------SLKTPLPGKLLPSAVVQ--PDWLPALMEHTRRIRAD   300
Lu_71Q2     MADAKELGVP-SYVFYTFSATFLGLMFQLQALYDEGRFNPVQIKDSDTEFVEIS----------SLKTPIPGNLLPSAVVE--PDLLLSLITHTRRTKEY   300
Lu_71M1     IDVANELGIP-SFLFFTSSIAFLGFMLYLPIRHD--RVGTGFELDDPAESVLVP----------SYATPISPRFLPSVLLDNRGGGYSTMTYHGRRFWE-   300
Lu_71R1     VDLGKELGVP-SYLFFPSCAAILGHLVHLPAMG----YSPEVAVGDPDGEVVYP----------SFEHPMPNRILPAIVLD--GQGYQELMGHTRKYNE-   300
At_88A1     LDITADFTFP-VYFFYTSGAACLAFSFYLPTIDE--TTPGKNLKDIP--TVHIP----------GVPP-MKGSDMPKAVLERDDEVYDVFIMFGKQLSK-   300
Lu_88A11    LSVADELGVP-GYQFSTSGAACLGFFLYLPTLHK--TTS-VSFKDLDNTDLEIP----------GVPK-LPSRDVPKILLDRDDVVYSYFLEFGTLLPK-   300
Lu_88G1     VSVADDIKIP-CYFYFTSCLYGLAIFLYFPVIHE--SSE-VSLKDVPDSLVPIP----------GLQS-IPSEDIPPAMADRGGRAYSGFISTAYNMVK-   300
Lu_72N1     FSVADEFGML-KYAFITTTASFLAVTVYGGVSEDEVV-EHVTLKKP----LHVP----------GC-NPIRFEDTLHAYLDYGDRVFDEAQKLGAGF-AL   300
Lu_72N2     FSVADEFGML-KYAFITTTASFLAVTVYGGVTEHEVV-EHVTLKKP----LHVP----------GC-KPIRFEDTLHAYLDYGDRVFDDAQKLGAGF-AL   300
Lu_72R1     FVIADEMGIG-KYLLGTSNAWFTALTLHTPALDKEVDGQYVDQTEP----LTIP----------GC-RLVRPDEVVDPMLDRNDMQYVEYKRIGAEF-AK   300
Lu_72R2     FAIADEMEIG-KYLLVTSNAWFTALALHTPALDREVDGQYVDQTEP----LTIP----------GC-RSIRPDEVVDPMLDRNDMQYVEYKRTGAEF-AK   300
At_72C1     LEVAKELGIMRKHVLVTTSAWFLAFTVYMASLDKQELYKQLSSIGA----LLIP----------GC-SPVKFERAQDPRK--YIRELAESQRIGDEV-IT   300
Lu_72Q1     FDICKDLHIP-VYSFFTASAALMTLSLYLPTMDRDIQGQFVYLPEP----VNVP----------GC-TPIRTHDLLDQVRNRNNDEYKWYLYHVARL-PL   300
Lu_72B15    FDVAAEFGAA-SYVFYPSTAMALSLFLYLPTLDAEVTGAYSDLDEP----VQIP----------GC-IPVNGTDLLDPVQDRNNDAYSWLLHHAKRY-RL   300
Lu_72B16    FDVAAEFGAA-SYIFYPSTAMALSLFLYLPKLDAEVTGPYSNLEEP----VQIP----------GC-IPVNGTDLLDPVQDRNNDAYSWLLHHAKRY-RL   300
Lu_72T1     FDVASEFGVP-GYVAMLSNAMLMSMVAHLPKLDEEVVGEYTDMKEP----ILFP----------GCRVAIHGSELPSPALNRKNDGYKWFLHNVKHM-DL   300
Lu_72T2     FDVASEFGVP-GYAAMLSNAMSMSMVAHLPKLDEEVVGEYTDMKDP----ILFP----------GCRVAVRGTELPSPALNRKDDGYKWFLHNAKQM-DL   300
Lu_72S1     FDVAREFKLE-SYFFVPSNVLTLALCNYMPKLDADVQGDYRQLTEP----IRLP----------GCRFVFPVEDLHPSILDRNSDAYPMLLRHSKRQ-RL   300
Lu_72M1     LQVASQLQIP-PYVFYTCSAFHLTLGLKAPELHQTHPEEFRDSSEP----LKLP----------GC-VPFPGPDLPDPYLDKKKDAYKWMLHVHERISND   300
Lu_72M3     LQVASQLQIP-PYVFYTCSAFHLTLGLKAPELHWTHPEEFRDSSEP----LKLP----------GC-VPFPNADLPDSYLDKK-DAYKWMLHVHERISAD   300
Lu_72M2     LQVATQLQIP-PYVFYTCSAFHLTLGLNAPQL--LHPTHQEDSTKL----LKLP----------GC-IPLLGADLPEPYIDKKKDAYKWMVHSHERISSD   300
Lu_90A8     LDSANKFGIP-RLVFYGISCYASCVCKSVGEG--KLLAR--ALSDHDPVTLP-----EFPWI----QVTKQDFEPPFDDPE-AKGAYFDFHLSCFISTAN   300
Lu_90A10    LESSIKYGFP-RLVFNGMCNYSICVFRSVVQS--GILFG--NELDDELIPVP-----KFPWI----KVTKNDFESHVKNPVGINGPDYEFVMKSMTASKS   300
At_90A1     SESAAKFNIP-RFVSYGMNSYSAAVSISVFKH--ELFTEPESKSDTEPVTVP-----DFPWI----KVKKCDFDHGTTEPE-ESGAALELSMDQIKSTTT   300
Lu_90D1     QDTAEKFGIP-RLTFYGMSNHAASVSRAVAID--RLLLG--PESEDELITVT-----QLPWM----KVCKNDFHEDSRSPE-PKGVNAEFIWKSVMASSR   300
Lu_97B2     LDTCRAFGIP-RLVFHGMSVCSLATIKSLWCAPPELKMMMMSPDKNQPLDLPNM---KLPFA----LTAADVPAEVMVNSSGEEDPLTKYIEEVGWADAN   300
Lu_97B3     LDSCRAFGIP-RLVFHGMSVCSMAISKSLWCAPPELKMMMTSADKKQPLDLPNM---KLPFT----LTAADVPAEVMVNSSAEEDPLVKYIEEVGWADAN   300
Lu_97B1     LDSCRAFGIP-RLVFHGMSVCSMAISKSLWCAPPELKMMMTSADKKQPLDLPDM---KLPFT----LTAADVPAEAMAPNANEEDLLAKYIEEVGWADAN   300
Lu_73W1     AHVAEKFGVP-RITFNGFSTFTLLCLR--YIH--DKNVMGVVGRDSEPFVVP-----GIPD---RVELTKNQLPLSMTDG-LDRFG-----EQIMVAEAL   300
Lu_73W2     AHVAEKFGVP-RITFNGLSTFTLLCLR--YIHV-DKNIMDAVGLDSEPFVVP-----GIPD---RVELTKNQLPLSMTDG-LDQFG-----QQLVVAEGL   300
Lu_73W3     SFLAQKHGIP-RISFNGFSSFAWLCICNMGISI-KEGFLNGVSSDCEPFLVP-----GMPH---RVELTNDKLPFDMIKG-MDQFN-----QRYEAAEAL   300
Lu_73W4     SFLAQKHGIP-RISFNGFSSFSWLCISNMWISI-KEGFLDGVASDCEPFLVP-----GMPH---PVELTNDKLPFDMIKG-MDQFN-----QRSEAAEAL   300
At_73C1     NRIAKNLGIP-KIIFHGMCCFNLLCTH--IMHQ-NHEFLETIESDKEYFPIP-----NFPD---RVEFTKSQLPMVLVAGDWKDFL-----DGMTEGDNT   300
Lu_73X3     NNVAKKFNVP-RISFHGFSCFNLFCMHCIRLHE-DEIVRTVGYSAHEYFVLP-----GLPG---EIKYTKVQMPIEIREPGNDDPKYEFHDS-VEKAESE   300
Lu_73X4     NKVAKKFDVP-RVSFHGIGCFSLVCLQCIIIHE-EELAR-MASSDHEYFVLP-----GMPG---EIKFSNAQLPLQIRKNGHEDPKEESPNHNAIKVDSE   300
Lu_73X2     NNVAKKFNVP-RISFHGFSCFCLACLHCMKLHE-AEVDL-SVSSDFDSFLIP-----GFPG---GIRFTKAQLP--LRGGGKDKEKNAEIAEEMKKAESD   300
Lu_73X1     ANLAAKFGIP-RISFHGFSCMCLLCVRLICLHA-DEIQK-DVPSDSDYFVLP-----KFPD--DRIRFTKLQLPMSVTKETKG------IGAQMLKVESE   300
Lu_73T1     VATSRKFGIP-RLVFDGMSCFAVACSHNILVS----KISETISSDRESFLVP-----GLP--D-RIRLTRAQLPVQFNS-SSLKLS--ELFDKMMEAEEQ   300
Lu_73V1     TEIASRLKIP-RLIFDGTSCFTLSCSHNLQVS----KVYEEVS-ESEPFVVP-----GLP--H-RVEFTRAQLSGLFNPGAHLDVS--EIREKISESVDK   300
Lu_73S1     ARLAERLGIP-RIVFHGMSCFSLLSALNIRKT----NAHLSSADEYEPFLVP-----GMPKCF-HVHVSRVQLPGSFVR--LPDLD--DVRNKMQEAETT   300
Lu_73B12    TDSSEKFGIP-RLLFHGTSFFSLTVMDVLSRY----EPHKDVSSDTEPFEVPG----GLPD---KIMLTKRQLPASAVATGQGDTFMWEFFKKVRESNSQ   300
Lu_73B13    TDSSEKFGIP-RLLFHGTSFFSLSVMDVVSRY----EPHKDVSSDTEPFEVPG----GIPD---RIMLTKRQLPASAVTPGQEDSFLWEFFERVSESNSH   300
Lu_73B7     TATAAKFGIP-RLVFHGMGFFALSVLASLATD----EPHRKVGSDSEPFLVP-----KLPD---EIFLTRRQLPEAEKE---EDEFLVSFFRDAKESEWK   300
Lu_73Y1     TASAAKYGIP-RLVFRGTSFFSSCVSEFITRY----KPHDAVSSDSEPFLVP-----GLPD---PVMVTRNQMPPPDKLT--SETFLGKVLKQIADSGKE   300
Lu_73Z2     TESAHRLGIP-RLFFNGTGSFSMCLIDCFKRY----DPCKGVESDSEPVVLP-----GLPH---KIEFKKSQLPPFWKGE-KVDDKIEELRHLIDKSEEE   300
Lu_73Z3     TESAHSLGIP-RLFFNGTGSFSMCLIDCFKRY----DPCKGIESDSEPVVLP-----GLPH---KIEFKKSQLPPFWKGE-KVDDKIEELRHLIDKSEEE   300
Lu_73Z1     TESAHSLGIP-RLFFNGTSSFAICLMHCFTRQ----EPWKGVESDSEPFVMP-----GLPH---RIEFTKLQLPPFWKGE-GITEEWLEMRDLINESEEK   300
Lu_73Z4     TETAHGLGIP-RLFFNGMGSFATCLFERLKES----DQYKKVESESDPFFMDI----GISN---RFRFTKMQLPPCLKGE-EVESRLVEFRDRIEESEAK   300
Lu_73Z5     TETAHGLGIP-RLFFNGTGSFAMCLFERLKES----DQYKKVESESDPFFVDI----GVSN---LFQFTKMQLPPCLKGE-EVESRLVEFRDRIEESEAK   300
Lu_73U1     ADIASDLGIP-RLAFSGSGFFNLCIADSIESN----NPHRRIQSETEEFVVP-----GIPD---LVKLTRSQLPDMVKGK----TEFSGFFDTLKQAERK   300
Lu_89B3     HHLASDLGIP-RIVFSPSAAFALSVIYHLWRNMP----QLPENPS-ESITFP-----DLPNS----PNWIKSQLSPIYRSYVPGDPQSELVKDGFLADID   300
Lu_89B4     HHLATDLGIP-RIVFSPSAAFALSVIYHLWRNMP----QLPESPD-ESITFP-----DLPNS----PSWIKSQLSPIYRSYVPGDPLSEFVKDGFLADID   300
At_89B1     KN----LGIP-RFDFSPSAAITCCILNTLWIEMPT---KINEDDDNEILHFP-----KIPNC----PKYRFDQISSLYRSYVHGDPAWEFIRDSFRDNVA   300
Lu_89E1     NQLASELGIR-HVLFSPSGAFAISVATSLWRDEP----LCPINDE-EIITFP-----TVPNS----PSYPWRQISFIYRMLEKGDPDREIFRDCFLANLS   300
Lu_89E2     NQLASEMSIR-HVLFSPSGAFGISVATSLWRDEP----PFPINDQ-EIITFP-----TVPNS----PSYPWRQISFIYRMLQKGNPDREIFRDCFLANLS   300
Lu_89D4     LSLSTRLGIP-RFAFFSSGAFLASLTDKLFRDPV-------AMRNLDCIVFD-----ELPGS----PSFKAEHLPSMFRRYVPDDPDWELVREGVLSNLV   300
Lu_92A3     APVAKEIGAF-HVIFSGSGGFGWACYYSIWLSLPHRNCDEETKGEYFRLEDFH-----------EASRFHKTQLPTSILEADGSDPWSLFQRENLTAWRD   300
Lu_92A4     VTVAKEIGAC-HVVFSGSGGFGLACYYSIWLSLPHRNCDEETKGGYFQLEDFH-----------EASRFHKTQLPTSILEADGSDPWSLFQRENLTAWSG   300
At_92A1     GKVCKEVGVY-SVIFSASGAFGLGCYRSIWLNLPHK----ETKQDQFLLDDFP-----------EAGEIEKTQLNSFMLEADGTDDWSVFMKKIIPGWSD   300
Lu_92G2     NDVAKANNTP-NLTFTTGGAYGTLAYISIWLNRPHKRADGQEEEEYFDVPGFG-----------DGRRFHITQLHQFLRKSDGTDSWSKFFQIQLCKSLN   300
Lu_94H1     EAAAESNVVGIKCVLFVPGGAACYSFLAHYSI--------KPAGAQYPLQDWGIAS-----------RE--GFLKKRATYPNGLRDEDLDTYTDCMKRS   300
Lu_94K1     QLASS--LLHIPAVLFLSTGAAMFAFAAHAYK--------FGRDNINNEASFPFASSIYL-----RDDREERAFVSRMLEPTCGNEINDHNWVQLCQERS   300
Si_94D1     EEAES---QNIPAMVFLSTGAAAISFIMYHWF--------ETRPEEYPFPAIYFRE------------HEYDNFCRFKSSDSG---TSDQLRVSDCVKRS   300
Lu_94G1     KFALS--LLKIPTVLFMTNAAFSTAVPLTSMK--------NTGKFDG---VFPLRS---------------NYIFDYEEVES----PSFKDRVFQSLERS   300
Lu_94G2     KLAFS--LLKIPTVLFMTIAAISTVVPFTSMK--------SSGKFDC---LFPLRS---------------NYFFDYEQVES----PSIMDRVFQSWERS   300
Lu_94G3     KLALS--LLKIPTVCFMTAPAIVYDFGFTSMN--------ASGQFNG---HFPMRS---------------NHVVDYGAAEST---PSLTDRFMRSMERS   300
Lu_94G4     KLALS--LLKIPTVCFMTSPAIVYDLAFTSMN--------ASPQFNG---AFPLRS---------------NYVIDYVEAEST---PSLANRVVWSMERS   300
Lu_91J1     AVARRLG---IPTVFFSIFIASSLAFSSPPADDEDEDYRRAAEDLAEKPRWVPFETTSG------YFRPFEAKASFEMVLVGGGGQEFPEIHRFRQMLRG   300
Lu_91J2     PIARRLG---IPTVFFSIFIASVLAFTSPPADDEDEDYRKTVEDLTEKPRWVPFETTSG------YYRPFEAKVSFEMAVVGGGGQEFPELHRFRQMLRG   300
Lu_91J3     PLARELN---IPTVYFSIFLASVMAFVGPPVG--EAEYREKTEDYTARPRWMKLNSTV-------YYRRFEAEKAFPAISGDGELRIVPEFHRFQQSLRG   300
At_91A1     PISRRLG---IKTGFFSAFNGATLGILKPPGF---EEYRTSPADFMKPPKWVPFETSV-------AFKLFECRFIFKGFMAETTEGNVPDIHRVGGVIDG   300
Lu_91C2     SAAADAGGGGIGCAFFSLFTATTLCFVGPPGG----DSRRNAEDFTVVPDWIPIEIKSNI-----AYRLHEVSKYVEKTDEDTSGP--SDQIRFAVAMEE   300
At_79B1     EIAKPIG---AKTVCFNIVSAASIALSLVPSA--EREVIDGKEMSGEELAKTPLGYPSSK-----VVLRPHEAKSLSFVWRKHEAIG-SFFDGKVTAMRN   300
Lu_79B20    DITASLG---IKSVNYTVLSAAAVAFADVP----VRGIVKGKELTVDELAVPPPGYPSST-----IVLRPHEGKLLSFIFFPYGEAT-TFWERISTGMRM   300
Lu_79B18    RAARELG---CVPVSYGTSSAVWAAMRIVP------SARIVKEMTDEELGRTPPGYPSSA-----VIPRPDEIAGARLFAMEFGPSS--LYERIVSVIQG   300
Lu_79B19    RAARELG---CVPVSYGTSSAVWAAMRIVP------SARIVKEMTDEELGRTPPGYPSSA-----VVPRRDEIAGARLFAREFGSSS--LYERIVAVIQG   300
Lu_79B17    SLGLELG---FKKVAYVTASAVSSALRILP------SVKMVKGMTDAELMRPPPGYPSSV-----VVPRLDEVDQARFLAEDFGGSAVPFYERLTASNSG   300
Lu_79A3     GMGSKLG---IKTLSFTVFSAVSTGYLTVP-----ARIEGGKIPTVEDMKRPPKGYPETP-----VDQMKTFTARDFTYVYKKFNGGLSVIDRVLDCRNA   300
Lu_79A4     SSGSRP--------------LASQFFLQFP-----PGTCG-KIPTAEDMKRPPKGYPETS-----VNQMKTFTARDFTYVYKKFKGGLSVID--------   300
            .......210.......220.......230.......240.......250.......260.......270.......280.......290.......300
```

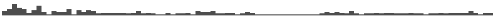

```
Lu_712B1   YHHVIVNSFHELEPSAFQLFP------------------NFLPIGPLVINSANSGGS--------------------FWRQDE-TCLTWLDN-------H   400
Lu_712B5   FDHVIVNSFHELEPSAFQLFP------------------NFLPIGPLVTNSTNSGGS--------------------FWRQDE-TCLTWLDN-------H   400
Lu_712B4   CDHVIINSFHELEPSAFQLFP------------------NFLPIAPLVTNSTNSRGS--------------------FWRQDE-TCLTWLDK-------H   400
Lu_712B3   FDHVIFNSFHELEPSVFQLFP------------------HFLPIGPLVTNSTNSGGS--------------------FWHQDE-TCLAWLDK-------H   400
Lu_712B2   FDCFIVNSFHELEHSAFQLYP------------------NILPIGPLVTNST-SIGS--------------------FWRQDP-TCLTWLDK-------H   400
Lu_712B6   FDCFIVNSLYQLEPAAFQLFP------------------KLLPIGPLVT-NSTSGGN-QH-------------NQIPGSFWHQDQ-TCSTWLDK-------H   400
Lu_712B7   FDCFIVNSFHQLEPTAFRMFP------------------KILPVGPLVITNSTSGGHHQY------------SQVPGSFWHQDQ-TCETWLDN-------Q   400
Lu_712D1   SDYMLVNSSQELEPSAFRLIP------------------NAFPIGPLQISTDIDPDDDTDN-----------SVLVGSLWPEDQ-TCLTWLNM-------Q   400
Lu_712C1   CDWILSNTFPELEPFACQLNP------------------DTLPIGPLLQTPDPTHFHG------------------NFWGAEDPTCITWLDQ-------Q   400
At_83A1    TDWLLCNSVHELETAAFGLGP------------------NIVPIGPIGWAHSLEEGS----------------TSLGSFLPHDR-DCLDWLDR-------Q   400
At_82A1    --WILTSSFKDEYEDVDNHKASYKKSNDLNKE-NNGQNPQILHLGPLHNQEATNNIT---------------ITKTSFWEEDM-SCLGWLQE-------Q   400
Lu_82A2    --CILVNSFQELDRDDDSNNPSLHKPQ------------RILQVGPLIEDQPR--------------------STISLWDHDS-TCLQWLDT-------Q   400
Lu_74S3    ADWVLCNSVHELEPKAADWLSKN--------------LPNFKTIGPTLPSFYLDKQ---------LPDDKDYGLSFFKPD--NEACSNWLQS-------K   400
Lu_74S4    ADWVLCNSVHELEPEAADWLSNH--------------LPNFRTIGPTLPSFYLDKE---------LPDDKDYGLSFFKPA--NEACSKWLQS-------K   400
Lu_74S5    ADWVLCNSVYQLEHEAADWLSKK--------------VPNFITIGPTIPSMYLDKQ---------LQDDVDYGFSIFKPI--NEPIKNWLSD-------K   400
Lu_74S6    ADWVLCNSIYELEPEAANWLSKK--------------VPNFTTIGPTIPSMYLDKQ---------LRDDVDYGFNIFKPI--NEPIKNWLST-------K   400
Lu_74S1    ADWVFCNTVYQLELEAVDWLTKQW-------------LINFRTIGPTIPSFYLDKQ---------IPDDKDYDISIFNPQ--NQTCMNWLQS-------K   400
Lu_74S2    ADCILCNTVYDLENETADWLSTI---------------WPLRTVGPTIPSMYLDKQ---------LQDDRDYGFSIFKPN--NEACINWLNN--N----K   400
Lu_74T1    ADCFLINTFYELEKEVVDTFSKI---------------CPILPIGPTIPSNYLTTKP-------SMTENGKYGLDLFEHD--ESIPIKWLSN-------K   400
Lu_74T2    ADYVLINTFYELEKEALHTMSKV---------------CPVLAIGPTVPSTYLDGR---------IDDDAEYGVDLFSLE--RSISTAWIAT-------K   400
Lu_74B3    VDWIFCNSFQELESKEAGSVKEH---------------WPAKLIGPMVPSSYLDSR---------IEGDNGYGASLWKPL--NDECTKWLEA-------K   400
At_74C1    ADCILCNTFDQLEPKVVKWMNDQ---------------WPVKNIGPVVPSKFLDNR---------LPEDKDYELENSKTEP-DESVLKWLGN-------R   400
Lu_74V1    ADWVFCNTFTSLEEKIVEWMGSK---------------LKFKTVGPTIPSMYLGKQQQQPHHDDTLEDHHEYGLSLFQPQS-PTRLVNWLNS-------Q   400
Lu_74V2    ADWVFCNTFSSLEGQVLEYLRSR---------------FKFMAVGPTIPSIYLS-----SNNGTKGAVSHDYGLSLFKPKPDEVDYMDWLDT-------K   400
Lu_74U1    ADWVFCNTFYTLEEKMLNWMTTQS--------------IQMKPVGPTIPSSYVG-------KEGPTQTNSNYGLSLFNPNSPQTSITQWLDS-------K   400
Lu_84G2    VFCILMETFQELEPE--IIRHVST-------------LQNN--IKPVGPLCLTGKISG------------------GDLMEVDDDCIKWLDG-------K   400
Lu_84G3    VFCILMETFQELEPE--IIRHVST-------------LHNN--IKPVGPLCLTGKISG------------------GDLMEVNDDCIKWLDG-------K   400
Lu_84G1    VLCILMETFQELEPE--VIRHLST-------------FFHDKMIKPVGPVCLAGKISG------------------GDLMEVDDNCIKWLDG-------K   400
At_84A4    PFSVLIETFQELEKD--TIDHMSQ-------------LCPQVNFNPIGPLFTMAKTIRS--------------DIKGDISKPDSDCIEWLDS-------R   400
Lu_75N1    NSKVLVNTFNELEAK--AINALDV-------------KFEMIGVGPLIPSTLVNRAQYSI----ANVNNRVLGINTAQEDKKKDCILTWLDT-------Q   400
Lu_75N2    KSKVLVNTFDELELK--AINASDV-------------KLEMIGVGPLIPSTLVNRVQYSI----VKVSNGVFGINTVQEDKEKDCTLTWLDT-------Q   400
Lu_75N4    KSKVLVNTFDELEME--AMKAN-V-------------ELEMIGVGPLIPSCFWE------------------PRHDNNNTDGGDSVVTWLDL-------Q   400
Lu_75N3    KSKVLVNTFDELEIE--AMKAN-V-------------ELDMIGVGPLIPSCFWE------------------PRDNNN----------------------   400
At_75C1    NPKILVNTFSALEHD--ALTSV-E-------------KLKMIPIGPLVSSSEGK---------------------TDLFKSSDEDYTKWLDS-------K   400
Lu_87J1    LQYLLFTSPYELEPKLFDALKTHF-------------QIPVYPIGPSIPHPKLAA----------------------ATG-RGIDYFDWLDA-------Q   400
Lu_87J2    LQYFLFTSPYELEPQLFHALKTHF-------------RVPVYPIGPLIPHLKLAA----------------------AAGGVEIDCFHWLDA-------Q   400
At_87A1    AKYLLFPSAYELEPKAIDFFTSKF-------------DFPVYSTGPLIPLEELSV----------------------GNENRELDYFKWLDE-------Q   400
Lu_87G1    AQYLLVNSVQDLEPESFDALKSEL-------------KLPIYPIGPAIPFYQLNHN-------------------NTNTSESAHAYFNWLES-------Q   400
Lu_87H1    AQYLLFTSIHELEVETMDSLSQTF-------------PFPVYPIGPAIPYLELLQN--------------------PTSS----SIHQWLNN-------Q   400
Lu_86A6    ADFILANTVQELEHDTISGLKQAH-------------KAQFYSIGPIFPPEFTTSS-------------------ISTSLWSESDCTEWLNS-------K   400
Lu_86A7    ADFILANTVQELEHDTISSLKQAY-------------NDQFYAIGPVFPPGFTISP-------------------VSTSLWPESDCTQWLNS-------K   400
Lu_86A8    ADFVLINTIQELEQDTISGLEHVH-------------EAQVYAIGPIFPRGFTTKP-------------------ISMSLWSESDCTQWLNS-------K   400
Lu_86A9    ADFILANTIQELEQDTLAGLKLAH-------------EAQVYAIGPIFPTEFTKSL-------------------VSTSLWSESDCTRWLNS-------K   400
Lu_86A10   ADFILANTVQELEQDTISGLKQAH-------------KGQVYSIGPIFPPRFTKSS-------------------VSTSLWAESDCTKWLNT-------K   400
At_86A1    ADFVVCNTVQELEPDSLSALQAKQ-------------P--VYAIGPVFS---TDSV-------------------VPTSLWAESDCTEWLKG-------R   400
Lu_76P1    --GLIWNSCEDLEQAALIKCRQ-------------VFKSPMFNIGPFHNYFP------------------------AALEEDQKNSISWLDT-------Q   400
Lu_76P2    --GLIWNTSQDLEHSNLLKSSK-------------LFKVPNFALGPFHKHFPCISK--------------------SSLLGEDLTSIPWLNS--N----Q   400
At_76B1    --GIIFNAIEDLETDQLDEARI-------------EFPVPLFCIGPFHRYVSASS---------------------SSLLAHDMTCLSWLDK-------Q   400
Lu_76N2    --AVIWNTMNCLENSLLSQVKQ-------------QCKVPIFTVGPMHKFSPPIS---------------------TSLLKEDYTCMPWLDS-------Q   400
Lu_76N3    --AVIWNTMNCLENSLLAQVKQ-------------QCKVPIFTVGPMHKFSPPIS---------------------TSLLKEDYTCIPWLDS-------Q   400
Lu_76N1    --AVIWNTMFCLEDSLLEQVRQ-------------RCSVPNFAVGPMHKFAPCLS---------------------SSLLAEDFSCMSWLDK-------K   400
Lu_76M1    --AVIWNTSTTLESSSLSIIHQNH----------TVPQIPIFPVGPFHKQILQPKT--------------------ETLTDEQTSALAFLDQ-------Q   400
At_85A1    ASAIILNTFDDLEHDVVHAMQ-------------SILP-PVYSVGPLHLLANREIE-----------EGSEIGMMSSNLWKEEMECLDWLDT-------K   400
Lu_85A22   PAAIILNTFDALEHDVLASLS-------------NMLAKPVYSIGPLQLLANDQTI-----------TDPSVKNISSSLWKEESECLDWLET-------K   400
Lu_85A25   VSAVIINTFHHLEQPVLDSLS-------------AIFP-PIYPIGPLTLMLDQIITPIPNPN----SNNNNLNSISSSLWKEEPECLQWLNT-------K   400
Lu_85A26   ASAIVLNTYDKLEEDVLRALS-------------RTLAPPIYTLGPLDLMTLREND---------------LDSLGSNLWKEESGCLEWLDQ-------K   400
Lu_85A27   ASALIINTFDKLERKFVESVL-------------PTFPP-IYTIGPLHLMDTRESA---------------LDSLGLNLWKEEHGCLEWLDR-------N   400
Lu_85R2    ASSVVFNTFEPLEQEALTYLS-------------SLCP-NLLTIGPLNSLLPRIIT------------EDKLKNINTNLWEEHPESVKWLDS-------Q   400
Lu_85R3    ASAVFVNTFDALEHEALSSLS-------------PLCP-NLLTVGPLN-LLNHQTT------------GDKLKSITTNLWTEHHESVQWLDS-------K   400
Lu_85R4    GSTLIMNTFDSLEKEALASLS-------------PLCP-NLLTVGPLINLLDQVK-------------EEKLNNIDANLWIEHPESLQWLDS-------Q   400
Lu_85R5    GSALIMNTFDSLEQEVLSSIS-------------TLCP-NLLSVGPLTNLLDQVK-------------EEKVKNINTNLWAEHPESLKWLDS-------Q   400
Lu_85R1    ASALLINTFDDLEHDALAALS-------------PLTP-NLFTVGPVNLLTPHITQN-----------KRVLENINANLWAEQSEWAGWLDS-------R   400
Lu_85K6    SGAIIFNTFDALEEQVLSAIKM------------DYYPQPIYTVGPLHLLGKEMLEP-----------ATESNSISSNLWKEDLGCMEWLGQ-------R   400
Lu_85K7    SSAIIFNTFDALEEQALASIR-------------KIFPNKMYTIGPHHLLGNEDDTD-----------DQSTRSISSNLWKEDLKCMDWLDR-------Q   400
Lu_85Q2    ASALVIHTYDAFEADVLAAIN-------------DLYPGRVYTIGPMQHLLNQIKQS---------TKLGLDDSIGYSLWEEEPECLRWLDS-------K   400
Lu_85Q3    ASALLLHTFEALEVDVLAALN-------------TMYPDRVYTAGPMQLLLNQAKHT---------SDL---DSISYSLWEEDSKCLRWLDS-------K   400
Lu_85Q1    ASAVLLHTFDALEPNVLTALN-------------EIYPNRVYPVAPMQLILNQIKST---------QQESSLDTISYSLWKEEPECLRWLDT-------K   400
Lu_85S1    APAIIFNIFEEFEDEIFFKIK-------------KFYP-HLYPIGPLSLLENHVVP-----------LDSPIRTHRTTLWKEDVECLDWLDT-------R   400
Lu_709E2   AYALILNTFEDLEGPILGRIR-------------TRCPKTYPIGPIHEQLRLKLVNLKTPQ--------ESSSSSSSSLWEEDRSCMKWLDL-------Q   400
Lu_709E3   VYALILNTFEDLE-----------------------------------------VEIKAFQP-------QNSSRIIIVVREEDRSCMKWLDL-------Q   400
Lu_709E1   LSPLILNTFEDLDSSVLSQIR-------------RHFPQTYAIGPLHQHLESRLRTMSFGSQ----NNINTQSSSSNSLWKEEASCLKWLDQ-------Q   400
Lu_709D1   ASGLILNTFDELEGSIISKLSS------------TIFPKTYPVGPLHG-------LLNNVVK---------EHHSDGGLWREDKGCMTWLES-------H   400
At_78D3    ATAVFINSFEELDPTFTNDFRSEFK--------------RYLNIGPLALLSSPSQT--------------------STLVHDPHGCLAWIE--K-----R   400
Lu_78A13   AAAVFINSFHALDPTITDDLSSKLN--------------NFLNIGPFHLLYPSPAS--------------------KEQQQQPSDCISWLNDQR-----H   400
At_71B1    TKGILVNSVADMEPQALSFFSGGNGNTNI---------PPVYAVGPIMDLESSGD-EE-------------------KRK----EILHWLKE-------Q   400
Lu_71P1    AKGIVINTFPELESATIEHLSKG-GN------------PPVYPVGPILELKRGGG-DV-------------------KDKGRSSDIMNWLNE-------Q   400
Lu_71A24   TKGILVNTVKEVESYAIDSLSRGLINN-----------PNIYTVGPILNLKEDTS-SS-------------------NS----NDVIQWLDE-------K   400
Lu_71A25   TKGILVNTVKEVESYAIDSLSRGLN-------------PNIYPVGPILNLKGDTS-SP-------------------SSSSGGNDVIQWLDE-------Q   400
Lu_71Q1    AKGILVNTFEDFESYAIASLNTGQSQT-----------PPAYPVGPIMDLKVKGGEST-------------------AAEHSVGPIMEWLDQ-------Q   400
Lu_71Q2    ASGILINTFQDFESHAIASLNAGQSQSQTP--------PPIYPVGPIMELKVKD------------------------ADHSAGPIMEWLDQ-------Q   400
Lu_71M1    TKGIIVNSFAELEPHAVKSIT----SLLLPGGA---LPPPLYPVGPLLDLKGQGQARFG------------------KTG-QRDEIMKWLDD-------Q   400
Lu_71R1    VDGIVVNSYVGLESRAINILNGKVDGVFRIGGK---SFPPVFPVGPVLNLKG--HATLG------------------NTKSLSEKAMTWLDD-------Q   400
At_88A1    SSGIIINTFDALENRAIKAITEELCFRN------------IYPIGPLIVNGR-----IED-----------------RNDNKAVSCLNWLDS-------Q   400
Lu_88A11   SAGLIVNSFDSVEEKAVKAISEGFCVPDGP-------TPPIYCIGPLIAAG-------DD-----------------RKSD-GGECMTWLDS-------Q   400
Lu_88G1    SAGIIVNTFELLEGNAFRAISEGRCTPGKS-------PPPIYCIGPIVEEK--------D-----------------KNGK--DACLTWLDS-------Q   400
Lu_72N1    ADGILINTWEDLEVQTLAALRSEKHLKN-IVK------APVYPVGPLVRP----SQP--------------------TGSTENNTVLEWLDE-------Q   400
Lu_72N2    ADGILINTWESLEVQTLAALRSEKHLKN-IVK------APVYPVGPLVRP----SPP--------------------TGSTENNTVLEWLDE-------Q   400
Lu_72R1    ADAILINTWEDLEPSTLAALRNDKFFGGSVIK------GDVLSIGPLVRP----SNNNQ------------------KGPTDDDELFSWLDK-------Q   400
Lu_72R2    ADGILINTWEDLEPSTLAALRNDKFFGRSIIK------GDVLSIGPLVRP----SNN-Q------------------RGPTEDDELFSWLDK-------Q   400
At_72C1    ADGVFVNTWHSLEQVTIGSFLDPENLGRVMRG------VPVYPVGPLVRP----AEP---------------------GLKHG--VLDWLDL-------Q   400
Lu_72Q1    AAGIFLNSWEGIEPVSIKAVKEHSFYKE-IPI------PPVFSVGPLIKQ----VEC-I------------------PLTDSDLDLLRWLDD-------Q   400
Lu_72B15   ADGVMVNSFPELEPGAIKSLQKTE--DQLGKK------PMVYPVGPLVN------MDSS------------------KKTG--SECLDWLDV-------Q   400
Lu_72B16   ADGVMVNSFPELEPGAIKSLQKTE--DQLGRK------PMVYPVGPLVN------MDSP------------------KKTG--SECLDWLDV-------Q   400
Lu_72T1    AEGVLINSFTDLEGETIRFLQKNM--NKP-----------IYPIGPIIQS-----GDSS------------------ITDP--SGCIKWLDH-------Q   400
Lu_72T2    AEGVLINSFTDLEGETIQFLQENM--NKP-----------IYPIGPIIQS-----SDGS------------------ISDP--NGCMKWLDN-------Q   400
Lu_72S1    ADGFIVNSFMEVEGEIIEALRGEE--FANGRP--------IFPIGPILQS-----TAAN------------------SSSGPTDECLEWLDK-------Q   400
Lu_72M1    AAGIMINSFMELESEIFKALTEER--SRTGSG------TAVYPIGPVPRLES--DEDLA------------------KLSNESIECLKWLDK-------Q   400
Lu_72M3    AAGIMINSFMELESEIFKALTEER--SRTGFG------PAVYPIGPVPRLES--DEDLV------------------KLSNESIECLKWLDK-------Q   400
Lu_72M2    AVGIIINSFVDLESDIFKALTEER--FRTGSG------PTVYPIGPLKRLDS--DEDLN------------------QFSNESIDCLEWLDK-------Q   400
Lu_90A8    SFGLIINGFYELEPLFVDHLN--RHA-----------LPKAWCVGPFFLAQPNKKGDETDHYLV----------KPYTKPT----WIEWLDRNL-----R   400
Lu_90A10   SYGYVVNSFYELEPVFVDSFNNFVSG-----------GPKAWCVGPLCLAKAHEK------------------IEHQKPS-----WIQWLDEKT-----E   400
At_90A1    SHGFLVNSFYELESAFVDYNNN-SGD-----------KPKSWCVGPLCLTDPPKQ--------------------GSAKPA----WIHWLDQKR-----E   400
Lu_90D1    SFGYVMNSFYELESVFVDYLNG--LG-----------SQKHHCVGPLCLADDENDAVG----------------NNKDENP----WMSWLDKKL-----E   400
Lu_97B2    SWGIIVNSFHEVELSHTESFEKFYFN-----------GAKTWCLGPLFLCE-GKKGTINP-------------NAHANSSAGSDELSRWLDEQV-----A   400
Lu_97B3    SWGIIVNSFHEVELSHTESFEKFYFN-----------GAKAWCLGPLFLCE-GKTGIIN---------------ANANSSTSWEELSRWLDEQV-----A   400
Lu_97B1    SWGIIVNSFHELELSHIEPFEKFYFN-----------EAKAWCLGPILLSHRVDHEMIN----------------PNTNS-----LSRWLDEQV-----A   400
Lu_73W1    SYGMIVNSFEELDPEYVEKYKVAMG-------------GKAWCVGPVSLVNESQLDRLQRGNN-------------AQYATGESKCLNWLDSR-------   400
Lu_73W2    SYGMIVNSFEELDPEYVEMYKVAMG-------------GKAWCVGPVSLVNESQLDRLQRGNN-------------AQYADGESECLKWLDLQ-------   400
Lu_73W3    SYGTIFNSFEELEHEYLSVFKGTMG-------------RKAWCVGPVSLCNKGEMDQFHRGN---------------QNSSDGSKCLKWLDSQ-------   400
Lu_73W4    SYGTIFNSFEELEHEYLSVFKGTMG-------------QKAWCVGPVSLCNEEKMDRFHRGN---------------KNSTDGSKCLKWLNSQ-------   400
At_73C1    SYGVIVNTFEELEPAYVRDYKKVKA-------------GKIWSIGPVSLCNKLGEDQAERGN---------------KADIDQDECIKWLDSK-------   400
Lu_73X3    VYGVIVNSFEALESEYFSGYKNSK-------------QGKVWCVGPVSLTNLHDLDKLQRGTS--------------DIS-LAHHSLDWLNTK-------   400
Lu_73X4    AYGVIVNSFEELEPEYFSKCKSSR-------------PGKIWCVGPVSLTNLNELDKIQRGHN--------------SIS-LTHQSLEWLNTK-------   400
Lu_73X2    AYGVIVNSFEELEAEYFELFKEAK-------------QGKVWCVGPVSLTNHDDLDKLQRGND--------------VTSNYLDECFQWLDTM-------   400
Lu_73X1    AYGVIMNSFHDLEEKYIAELKKGNGG-----------NGRIWCAGPVSLTNSDELDKLQRGGG--------------EGD--GRELVGWLDLK-------   400
Lu_73T1    SFGRIVNSFEGLEPAYVEMNR--RQ------------SKKVYCIGPVSLRNRNNSDRAMRVS--------------NKSGIGETECLKWLDQW-------   400
Lu_73V1    AYGVVFNSFEELEPEYVTECRKIRG------------ERKIWCVGPASLCNTDDPDKAERG---------------NKPSSDERNCLRWLDSW-------   400
Lu_73S1    SFGVVANTSEELEDGCAQEYQN-AI------------GKKVWCIGPVSLRNTHNLDKFDRG---------------NKPSIDQSLVLEWLGQR-------   400
Lu_73B12   GYGTVVNSFYELEPGYVDYYRNVFQ-------------RKAWHIGPVSLCNADVDDKANRG---------------KESSIDWDYCLNWLDSK-------   400
Lu_73B13   GYGTVVNSFYELEPGYADYYRNVLG-------------RKSWHVGPVSLCSADVDDKANRG---------------KESSIDREHCLNWLDSK-------   400
Lu_73B7    SFGVIVNSFCELEPTYVEHYRNTLG-------------RKAWHIGPLSLS----RQAYRG----------------NEDSIEAHDCLKWLDWK-------   400
Lu_73Y1    SYGSVNNTFHELEPAYADLYNEILGE-----------KKKVWSIGPVSLCNNEVKDRANRGG--------------KESSIDEDSLLQWLDSK-------   400
Lu_73Z2    SFGAVVNSFHELEPGYSEHYREVIG-------------RKAWFVGPLSVCNKDTTLDKADRG--------------DAAAIDGRQCLRWLDGR-------   400
Lu_73Z3    SFGTVVNSFHELEPGYSEHYREVIG-------------RKAWFIGPLSVCNKDTTLDKADRG--------------DAAAIDGHQCLRWLDGR-------   400
Lu_73Z1    SFGAVVNSFHELEPGYSEHYKEVVG-------------RKAWFIGPLSLSNKDSTLEKAERG--------------KTAAIDGHECLRWLDCR-------   400
Lu_73Z4    SYGVVVNSFHELEAEYAEYYRNVIG-------------RKAWFVGPVSLIDNNNVMDQA--------------------AIDGGKCLKWLDSK-------   400
Lu_73Z5    SYGVVVNSFHELEAEYAEYYRNVIG-------------RKAWFLGPVSLIDNNNVMDQA--------------------AIDGGKCLKWLDSK-------   400
Lu_73U1    SYGVLMNSFQGLESDYADHFKQFIG-------------LKAWQLGPVSLFVNRINLDVDKFNSG---------GKAAADVITGDKFLNWLDSE-------   400
Lu_89B3    SWGIAFNSFAGLESKYLEYLKIELG------------HDRVWAVGPLLSPPS----ESVASRGG---------T----SSVSVPHLEAWLDTC-------   400
Lu_89B4    SWGIAFNSFAGLESKYLDYLKIELG------------HDRVWAVGPLLSPPS----ESVASRGG---------T----SSVSVADLEAWLDTC-------   400
At_89B1    SWGLVVNSFTAMEGVYLEHLKREMG------------HDRVWAVGPIIPLSG----D---NRGG---------P----TSVSVDHVMSWLDAR-------   400
Lu_89E1    SWGIVINTFARIEQPYIDHLKRESS------------HSRVWAMGPLLPPPS--GGGSSGDRGG---------A----SSIPSDQIRTWLDSR-------   400
Lu_89E2    SWGTVINTFARIEKPYIDHLKRESSS-----------HGRVWAVGPLLRPPSSGGGGGNSDRGG---------A----SSIPSDQIITWLDSR-------   400
Lu_89D4    SHGCIFNSFQALEGPSFDFLKGKMG------------HENVFAIGPVSMFGI--------DRNP---------N----SSSS--NVVEWLEHC-------   400
Lu_92A3    SDGILFNTVEEFDSIGLCYFRRKLG-------------IPAWAIGPVLL---------------------NRNRSNSGSGISSNSCKAWLDT-------K   400
Lu_92A4    SDGILFNTAEELDSIGLCYFRRKLG-------------IPAWPIGPVLL---------------------NRNLSNSGSGISSNSCKAWLDT-------K   400
At_92A1    FDGFLFNTVAEIDQMGLSYFRRITG-------------VPVWPVGPVLK---------------------SPDK-KVGSRSTEEAVKSWLDS-------K   400
Lu_92G2    SHGWLCNSVEEIEPLGFELLRKYTN-------------RQIWGIGPLLPPQFLLGSSSSS----------SRRTTAKTHGVSPEKCLEWLQL-------H   400
Lu_94H1    SDIILIKTSRNIEA-KYIDYLSELLG------------KEVVPVGPLVNDKPEDHRT-------------------GAEGDDDNKILKWLN------SID   400
Lu_94K1    CSIILIKTFREIEG-KYLDYISELAG------------KRHVPVGPLLQKT-----T-------------------SSEEDGGRRISKWLD------AKQ   400
Si_94D1    HDLVLIKTFRELEG-QYVDFLSDLTR------------KRFVPVGPLVQEVG------------------------CDMENEGNDIIEWLD------GKD   400
Lu_94G1    SPILLVKSFREIEA-EYIDTVSELFG------------KSVLPIGPLVPDDDDE-----------------------NQQPDEE-IINWLN------NKG   400
Lu_94G2    AGILLVKSFREIEA-EYIQRLSELVG------------KSVLPVGPLVPGDDDE-----------------------NQQPDND-IINWLN------NKA   400
Lu_94G3    STVVLIKSFRDIEA-HYIDRVSKFIG------------KSMVTIAPLVPDDDDG--E-------------------GDQEPDNNEIINWLN------KKH   400
Lu_94G4    STMVLIKSFRDIEA-RYIDRVSKFIG------------KSMVTVAPLVPDDDDDD-E-------------------GDQETDNN-VINWLN------NKD   400
Lu_91J1    CDYIAVRTCPELEY-DWLKLLEQMH------------KKPVFPIGVLPNPIKEE------------------------DEKEEGDEDTWKSIKEWLNDGK   400
Lu_91J2    CDFVAVRTCPELEH-DWLKLLEQIH------------KKPVFPIGVLPNPIKEE------------------------D--EEGDEDTWKSIKEWLDDGK   400
Lu_91J3    CDLIAVRSCREIEL-EYLDLLEEIQ------------GKPVFPIGVLLSEDNSG------------------------SEDSISGGSDWLGIRKWLDEHK   400
At_91A1    CDVIFVRSCYEYEA-EWLGLTQELH------------RKPVIPVGVLP-PKPDE------------------------K---FEDTDTWLSVKKWLDSRK   400
Lu_91C2    SNALLVRSSREFEP-EWFELLGQMYK-----------EKTIIPVGFLPPPIAAN------------------------D-KEDQNDAVWREIRDWLDKQR   400
At_79B1    CDAIAIRTCRETEG-KFCDYISRQYS------------KPVYLTGPVLPGSQPN------------------------QPSLDPQWAEWL-------AKF   400
Lu_79B20   CDALAIRTCDEIEG-KLCEYLGEQYK------------KRVFLTGPVLTEPAND------------------------VVSLENQWIDWL-------GKF   400
Lu_79B18   SEAIAMRSCRELEG-KYLDYLGEQHG------------KRVLLTGPVLPKLDG--------------------------LGLDEKLGCWL-------SKF   400
Lu_79B19   SEAMAMRSCRELEG-KYLDYLGEQHG------------KRVLLTGPVLPKPDG--------------------------LGLDENLGSWL-------SKF   400
Lu_79B17   GDAIAFRTCRELEG-QFCDYLGQQYG------------KPILLTGPILPDEDKTP-----------------------MTAEDEKLFSWL-------GNF   400
Lu_79A3    ATALVFKSCNEIEGSPYLDYLRTQFA------------KPVLSCGPLVPEPPTG--------------------------RLEDRWADWL-------GRF   400
Lu_79A4    --SLGLSQCRHRTGVQVMQ---------------------------------------------------------------RDR---------------   400
           .......310.......320.......330.......340.......350.......360.......370.......380.......390.......400
```

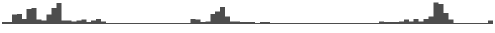

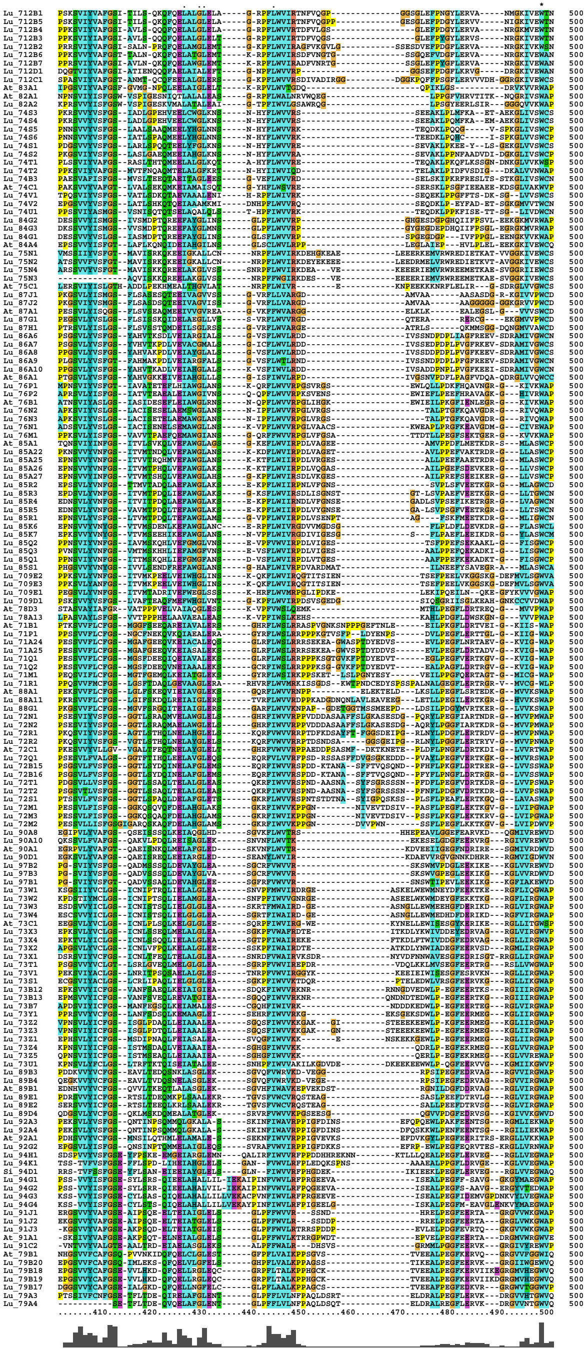

410
420
430
440
450
460
470
480
490
500

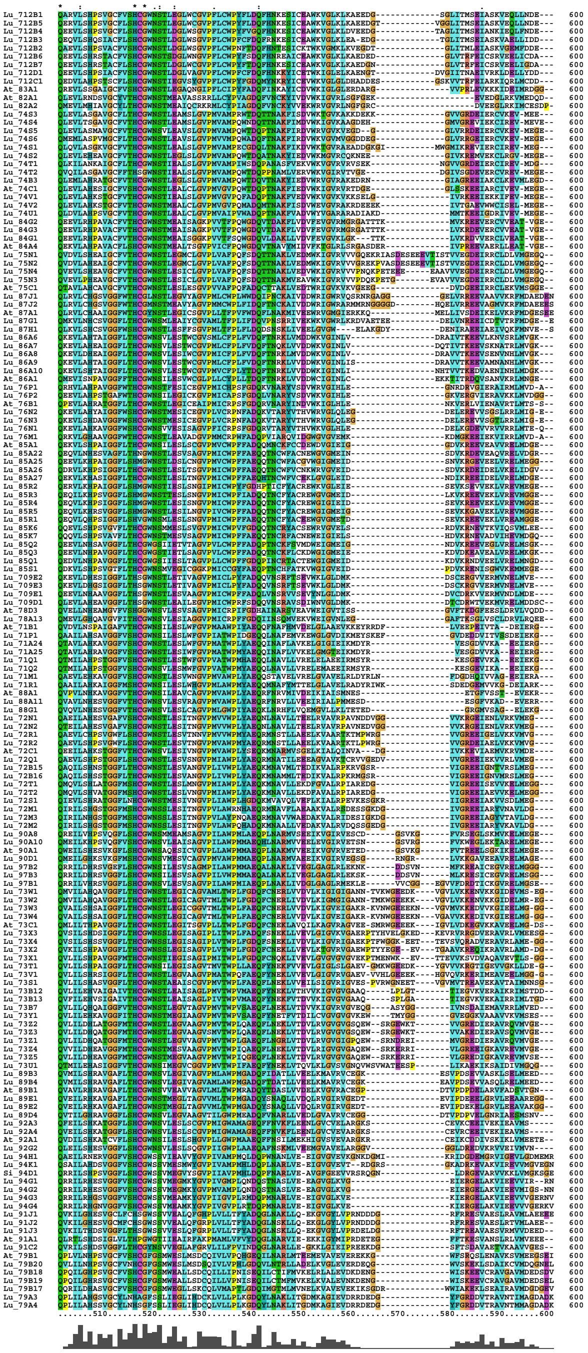

510
520
530
540
550
560
570
580
590
600

```
Lu_712B1   ----TIKGNA--------NRLREVARESVN----QGGSSFHSFSSFVNQLCSNDVACE-----------------------   681
Lu_712B5   ----TIKGNA--------NRLKEVARGTVN----QGGSSFHNFLSFVNQLRSTDVVCE-----------------------   681
Lu_712B4   ----IIKSNA--------IMLRGLARATVN----KDGTSFRSFMSFVDNLCS-----------------------------   681
Lu_712B3   ----TIRNNA--------NKLREVAQSSVN----KDGTSFHNFSSFIDNLSS-----------------------------   681
Lu_712B2   ----SIRDNA--------NGLMGMATESVN----EGGSSFCNFQKFINKLCS-----------------------------   681
Lu_712B6   ----TIRENA--------SKLRENARECVS----DGGTSFRNFLSFVEILCS-----------------------------   681
Lu_712B7   ----TMRENA--------SKFREQARECVS----EGGNSFRGFLRFVETLCS-----------------------------   681
Lu_712D1   ----TIKSNS--------MKLREIARDATCGSTTDTGSSFLKFETFVTELCNTLCKCHIDNVQEISKDYKRQLNLNGQDVC   681
Lu_712C1   ----GIKANV--------VRLKEMAVKSLS----PGGSSSTNLHTFIQQLC------------------------------   681
At_83A1    ----EYEERA--------MKVKEIVMKSVA----KDGISCENLNKFVNWIKSQVN--------------------------   681
At_82A1    -----MGERL--------RKLRDRAMGNEAR-----LSSEMNF-TFLKNELN-----------------------------   681
Lu_82A2    -GNGEMSCRL--------DKLCEMSTGKVAN-----LKRMANLNSFVDLVMDKC---------------------------   681
Lu_74S3    --GEETRRNC--------DKFAKLCKDAVG----ECGSSCRSITLFADSLILASRTHP-----------------------   681
Lu_74S4    -KGEEMRRNA--------EKLKKLMKDAVS----ESGTSHRNITEFAA---FCNHRN------------------------   681
Lu_74S5    -KGEEMRRNC--------EKFGELVKDAAS----EGGTSDGNIRRFAGSLIRAVKP-------------------------   681
Lu_74S6    -KGEEMRRNC--------EKFGELVKDAAT----EGGSSDGNIRRFASSLIRAVKF-------------------------   681
Lu_74S1    ---EETRRNA--------DKWGKIIKEAVV----EGGSSDKNTEDFATSLINFAETFQFSC--------------------   681
Lu_74S2    -EGKEMKRNA--------DKWRKMMKEAAG----EGGSSDRNISDFVDSLRNHRI--------------------------   681
Lu_74T1    -TGMAMKKNA--------TKWREAVVKAVG----KGGSSFRNIDDFVAKITTHKKTFETRMLIG-----------------   681
Lu_74T2    -KGKEMRSNC--------EKLKGLACLAIS----EGGSSDKSIDEFVSKLMSYKSH-------------------------   681
Lu_74B3    -RSKEIKEAA--------RKWRRMAVEAVS----EGGDSDKEIDRFVKHLMKGRS--------------------------   681
At_74C1    -RGKEIRKNV--------EKLKVLAREAIS----EGGSSDKKIDEFVALLT------------------------------   681
Lu_74V1    -KANGIRMNA--------EKWKSLAQAAVA----NGGSSDNNIQEFVAQLTNCQLLQSF----------------------   681
Lu_74V2    -RGKGIMGNV--------EKWKNLARAAVA----PGGSSDRNIDEFVAQLKSDNF--------------------------   681
Lu_74U1    -SGDKIRRNA--------KKWSALAKEAIG----DRGSSERNVQEFVTALVHGRLA-------------------------   681
Lu_84G2    -KAEMLRRNA--------ARWKKEAEAAVA----EDGSSTRSLLEFVEEVKKRNGTTS-----------------------   681
Lu_84G3    -KAEVLRRNA--------MRWMKEAEAAVA----EDGSSTRSLLEFVEEVKKRNGATL-----------------------   681
Lu_84G1    -KAEVLRRNA--------TRLKKEAQAAVV----KDGLSTRSIVEFVEEVKKRSFL-------------------------   681
At_84A4    -KAVELRENA--------RRWKEEAESAVA----YGGTSERNFQEFVDKLVDVKTMTNINNVV------------------   681
Lu_75N1    -VREQIRKNA--------NKWKQLAMDALR----EGGSSQSNLQAFVNEVYQ-----------------------------   681
Lu_75N2    -VREQVRRNA--------NKWKQLAMDALR----EGGSSESNLQAFVNEFTGK----------------------------   681
Lu_75N4    -VREQVTTNA--------DKWKQLARDALR----EGGSSHSNIKAFVDQIIGK----------------------------   681
Lu_75N3    -VREQVRTNA--------NKWKQLARDALR----EGGSSHSNIKAFVDQIIGKRL--------------------------   681
At_75C1    -EAEEMRENA--------EKWKAMAVDAAA----EGGPSDLNLKGFVDEDE------------------------------   681
Lu_87J1    DEVREMRKRA--------LGLGEDCRAAVA----VGGTSNLTLDSFVRDISK-----------------------------   681
Lu_87J2    GEVMEMRKRA--------SELREVCRAAVA----IGGSSDLSLDCFLKDICK-----------------------------   681
At_87A1    G--KEMRRRT--------CDLSEICRGAVA----KGGSSDANIDAFIKDITKIV---------------------------   681
Lu_87G1    SEVKEMRKRG--------KELREVCRGAIA----EGGSSDKNLDEFIKEISGAS---------------------------   681
Lu_87H1    EEVGRMRRRA--------REVGDICNGVTK----DGGSSVSNLDAFITGISQAV---------------------------   681
Lu_86A6    -TRDELQEKI--------KVVKKILVDALE----PSGSSEQNLARFVRELKDTILYRNQ----------------------   681
Lu_86A7    -SRDELKERI--------KEVNRILVDALE----PNGSSKQNLVRFIRELKDMIIYRHQ----------------------   681
Lu_86A8    -SRNELKEKI--------NEVKKILVWALE----PSGSSEQNFIRFIGELKDKII--------------------------   681
Lu_86A9    -SRNELKERI--------NGLQKILVDAIK----PSGSSKQNFARFVRELNDTIIQRKESW--------------------   681
Lu_86A10   -SRERIKEKV--------KEVNKILVGAIE----PNGSSERNFTRFVRELEDNITQLSK----------------------   681
At_86A1    -TSSELRNNV--------EKVKRHLKDAVT----TVGSSETNFNLFVSEVRNRIETKLCNVNGLEISPSN-----------   681
Lu_76P1    -EGQEIRQRS--------IALKDKIDDSLKQG----GSSYRSLDSLVSSILSAC---------------------------   681
Lu_76P2    -EGEEIRARV--------GDLKEKMEVCVKIG----GSSYEAVDQLVHHILGFCISGGGEASN------------------   681
At_76B1    -EGEEIRKRI--------MPMKETVEQCLKLG----GSSFRNLENLIAYILSF----------------------------   681
Lu_76N2    -EGDEIRRRA--------EEVRGKVEVETRRG----GSSVKDLDELVNMIRSF----------------------------   681
Lu_76N3    -EGDEIRRRA--------EKVRGEVEVETTKG----GSSVKDLDELVNMIRSF----------------------------   681
Lu_76N1    -EGTEIRKTA--------LELRKAVENSTIKG----GSSFNDLENLFDMIRSF----------------------------   681
Lu_76M1    -DGEGIRKNA--------LELKKKVLGSLAEG----GSGFDGLNQLVEFIGSC----------------------------   681
At_85A1    -KGKKMREKA--------VEWQRLAEKATEHKL---GSSVMNFETVVSKFLLGQKSQD-----------------------   681
Lu_85A22   -KGGEMRKKA--------AEWKRIAAEAVTGGG---GSSFAGLDSLIERVLLSSSRK------------------------   681
Lu_85A25   -KGKEMKLKA--------EEWKKLAAAAAQPG----GSSRRSFDELVELLQGRGGSKLAVEE-------------------   681
Lu_85A26   -KGKEMKETA--------MEWKRLAEEAAQCEI---GHAYLNLESVINNVLLNSV--------------------------   681
Lu_85A27   -KGKEMKRRA--------MEWKKSAEDATLGES---GLAYLNLEDMINNILLHNVKKN-----------------------   681
Lu_85R2    -KGKEMKRKA--------MEWKVKVDEATQPG----GSSFQNFDRFIGVLLQNKNN-------------------------   681
Lu_85R3    -KGKEMKRKA--------MEWKVKAEEATQPG----GSSFQNMERLIEVLLHNEDD-------------------------   681
Lu_85R4    -KGKEMKRKT--------MEWKVKAEEATNSD----GSSFQNLEKLIEILLQK----------------------------   681
Lu_85R5    -KGKEMKRKA--------MEWKLKAEEATQPG----GSSFRNLDKLIEILLQNTTN-------------------------   681
Lu_85R1    -KGKEMKRKA--------MEWRLKAEEATQPG----GPSFRNVERLIQVLLQKK---------------------------   681
Lu_85K6    -NWKMMKQKS--------VEWKTRAKDAVSEQ----GSSFNNFTRFFQDHLQYQS--------------------------   681
Lu_85K7    -KGEMMKRKA--------SEWQLKAREAVGVQ----GSSFTNFTSFLQHHM------------------------------   681
Lu_85Q2    -KGDKMRNKA--------MDWARLARESTGPG----GSSTVGLDRLVNEVLLKKPLVGI----------------------   681
Lu_85Q3    -NGDKMRNKA--------RDWARLAREATESG----GSSTVGFDRVINEVLLKK---------------------------   681
Lu_85Q1    -KGKKMRSKA--------QDWAKLAREATSPG----GSSVLNLDRLVSQVLSPNSK-------------------------   681
Lu_85S1    -DGKRMKNKA--------LEWKKKAEVATDIG----GSAYESFNRVLNVLNSSLE--------------------------   681
Lu_709E2   -RKEEFGRCA--------AKMAELAGMSVSSD----GCSSRNLEDLIEEIRSMRSS-------------------------   681
Lu_709E3   -RKEEFGRCA--------AKMAELARMSVSTG----GCSSRNLEDLIEEIRSMRSS-------------------------   681
Lu_709E1   -RKDEFQSLA--------AKMAALAKGSVSEG----GSSCRNLEVLIQDIRLMSVKGT-----------------------   681
Lu_709D1   -KRDEIVKST--------AEIARLARDSIKEG----GSSYCNLEKLIADVGAMC---------------------------   681
At_78D3    --GKKMKVNA--------KKLEELAQEAVSTK----GSSFENFGGLLDEVVNFG---------------------------   681
Lu_78A13   --GKKVRVNT--------RHLREKAEEATQSK----GSSSADFFKLVELVSKST---------------------------   681
At_71B1    IKCAME-QDSK------MRKRVMEMKDKLHVALVDGGSSNCALKKFVQDVVDNVP--------------------------   681
Lu_71P1    LKGLMEDQGGE------VRERVRKLSDKCREALAEGGSADIALNGFITDAIRS----------------------------   681
Lu_71A24   IRSLMDKDCG-------LKKKVEELRDRIREAFVDGGSSSSSIAQFIQDL-------------------------------   681
Lu_71A25   IRSLMDKDCG-------LKKKVEELRGRIREAFADGGSSSSSIAQFIQDL-------------------------------   681
Lu_71Q1    MMRLMSEESGGE-----RRKKVKEMSEKSRKTIVNGGASYYAISRFVEDVSKNIE--------------------------   681
Lu_71Q2    IMGLMSEESGGE-----RRKKTKEMSEKSRKTVENGGASYHSIGRFVGDD-------------------------------   681
Lu_71M1    VRSVMEEESA-------VRKKVKEMAGKCRAAIVDGGSSFAAIGSFLSGAILNS---------------------------   681
Lu_71R1    IEMVMDKHSE-------VRKKVKEMSELGRRALNEGGSSFDGIDGFMDLVLKNKPMV------------------------   681
At_88A1    VQEIIG-ECP-------VRERTMAMKNAAELALTETGSSHTALTTLLQSWSPK----------------------------   681
Lu_88A11   VKELME-SEGRGEL---VRRQTIKMKNEARSAVAEGGSSRVALSQLVDSWRSK----------------------------   681
Lu_88G1    VVELMSPESEKGKA---VRERVAAMQEGAAAAMSDGGSSRVAISKLVDAFKRE----------------------------   681
Lu_72N1    EEGQCIRERV----------KEVMEDGGSALSRKLNGSSFRALEKVAGECELNHRRVNAKAQGA-----------------   681
Lu_72N2    EEGKGIRERV----------KEVMEDGGSALSRKLNGSSFRALEKVAGECEWNHRRVKAKAQGA-----------------   681
Lu_72R1    EEGVLIREKV----------NEVKCSGEKALK-EGSGSSFKALASVVDKCACRYATKF-----------------------   681
Lu_72R2    EEGVLIREKVNEVKWSGEKVNEVKCSGEKALK-EGSGSSFRALASVVDKCTCRYATKF-----------------------   681
At_72C1    EEGKEMRKNV----------KELKKTAEEALNMTHIPSAYFT---------------------------------------   681
Lu_72Q1    EKGKLLRKKA----------KLLKKS--AALSLNDGGDSCESLAKVVRGWKLM----------------------------   681
Lu_72B15   EEGKKVRYRM----------KELKDAAKKVLSKDGSSS--RALSEVVQKWKDQKV--------------------------   681
Lu_72B16   EEGKKVRYRM----------KELKDAARKVLSKDGSSS--RALSEVVQKWKDQKV--------------------------   681
Lu_72T1    DQGAAVRKRM----------EKLKLAAAEAVGDEGSST--KSLAELVAKWTKGINN-------------------------   681
Lu_72T2    EQGAGVRKRM----------EKLKVAAAEAVGDEGSST--KSLAELVAKWIKGINS-------------------------   681
Lu_72S1    EEGAAVRRRM----------SELRKAA---LNAQVSVD--GSLEQLVLRWRKNPVE-------------------------   681
Lu_72M1    EEGKLLRRKV----------KELKAAANTAIGNDGSST--KSLDEVANLWKIQNP--------------------------   681
Lu_72M3    EEGKLLRRKV----------KELKAAANTAIGNDGSST--KSLDQVANLWKNQNHP-------------------------   681
Lu_72M2    DEAKLLRKKM----------RELKVAANNATGNDGSST--KSLDEVANLWKNQNPY-------------------------   681
Lu_90A8    -KGKEVRKRA-----------KEYGEMARKAMEEGSGSSWRNLDLLLGEIFTAKI--------------------------   681
Lu_90A10   -KGEEAKKKV-----------MEYSTKAMQAMGEKTGSSWRTLDMLIEELCRKRT--------------------------   681
At_90A1    -TGKTARKNV-----------KEYSKMAKAALVEGTGSSWKNLDMILKELCKSRDSNGASE--------------------   681
Lu_90D1    -KGKEVRKNV-----------EAFAEKAIKSMEKGSGSSWRTLDGLVRELWSYKAIAEK----------------------   681
Lu_97B2    -KGRHAKERA-----------QALGRVAHKAVQKG-GSSHEAMSRLVNELRQC----------------------------   681
Lu_97B3    -KGRHARERA-----------QALGRVAHKAVQKG-GSSHEAMSRLVSELRQ-----------------------------   681
Lu_97B1    -KGRRARERA-----------QALGRVAHRAVQRG-GSSDETMSRLISELRQC----------------------------   681
Lu_73W1    EEGDMRRRRV-----------KELSGKSKLALLEGGSSYV-NIERLKQDILEQTSIKLFTRH-------------------   681
Lu_73W2    EEGDMRRKRV-----------KELSEKSKLALLEGGSSYV-NIERLKQDIMEQTSIKSCKKNTVGL---------------   681
Lu_73W3    DERDKRNIRV-----------KELSEMAKLALQDGGSSFM-NIEMLIEDISRYESGF------------------------   681
Lu_73W4    DERYKRKIRV-----------KELSEVAKLALKPGGSSYM-NIEMLIEDISRYEVGF------------------------   681
At_73C1    NDAKERRKRV-----------KELGELAHKAVEEGGSSHS-NITFLLQDIMQLEQPKK-----------------------   681
Lu_73X3    EEGDGRRNRA-----------KELAEMAKRAMESGGSSHR-SVGMLIDDIMKHQEEKEYCKKL------------------   681
Lu_73X4    EEGDGRRKRA-----------EELAGIARTAVERGGSSYK-NVDVLIEDIAKHQEERRNHG--------------------   681
Lu_73X2    EEGGERRKRA-----------NEFGEMARRAVGIGGSSYR-NVGVLIDDVVQWHQERKLEGDLY-----------------   681
Lu_73X1    AEGEERRRKA-----------VEIAKMARHAVKNGGSSYE-DITRLIEEIKTFHVA-------------------------   681
Lu_73T1    EKGRERRKRA-----------RKLSKMAMESVEEGGSSYL-NIGNLINDVV------------------------------   681
Lu_73V1    DESEGRRTKA-----------EKLKGMATAAVADGGSSSK-SVGMLIEDVANFGGEFGV----------------------   681
Lu_73S1    EEGKKRKNRI-----------KKLAEMTNKSMGDTGSSHL-NLTELIADVVKQQGATQHALAKHVPSNRYDELRS------   681
Lu_73B12   EEVAKMRSRA-----------KYLGHMARKAVEEDGSSYC-DLDALIEELR---SNCRA----------------------   681
Lu_73B13   EEVEEMRRRA-----------KNLGEMARKAVEKGGSSYR-DLDALIEELR----LNRKA---------------------   681
Lu_73B7    -EGEEMRRRV-----------KMLGKAAAEAVE-GGSSWN-DLDNLVLELQSLSPMNRCSLL-------------------   681
Lu_73Y1    AAAVEMRRRV-----------AELGKMARRSVEEGGSSFG-NLGELIEEVKRCRIDNF-----------------------   681
Lu_73Z2    -HAEEMRERA-----------MELKEKAVKANEEGGSSYT-DLKSLLEELASVREKKDVDFSSLKL---------------   681
Lu_73Z3    -HAEEMRGRA-----------KELKEKAVKANEEGGSSYT-DLKSLLEELASVRDKKDVDLSSLKL---------------   681
Lu_73Z1    -HAEEMRERA-----------MELKVKAVKGNEEGGSSYS-DLKSLLKELASVRDKKDVDLSSCKL---------------   681
Lu_73Z4    -DVRKMRMRA-----------AELKESAKRADEEGGSSHC-DLKSLLEELSSLKGKIN----------------------   681
Lu_73Z5    -DDQEMRMRA-----------AELKELARRANEEGGSSYC-DLKSLLEELRSLKDKIN----------------------   681
Lu_73U1    PQAIEMRKKI-----------QKLAEMAKNAVEIGGSSDC-DLKSLLNDIRDYKKRKHNRPLNGYVDEV------------   681
Lu_89B3    ---REERKLA-----------KELSLAAKEAVSEGGSSVK-DMESLVEQLVQLYSSSD-----------------------   681
Lu_89B4    ---REERKVA-----------KELSLAAKEAVGEGGSSVK-DMESLVEQLVQLNSTSSD----------------------   681
At_89B1    ---QTERIKA-----------VELRKAALDAIQERGSSVN-DLDGFIQHVVSLGLNK------------------------   681
Lu_89E1    --VLSERERA-----------EELRTAARDALVEGGSSFT-DLDEFVEKLNGKD---------------------------   681
Lu_89E2    --MWWKRERA-----------KELRTAARDAVVEGGSSFK-DLDEFVEKINGKTTNSVLHRD-------------------   681
Lu_89D4    ---LGERKGA-----------EELKKKALTAIEEGGSSRI-DLDRLVHQLHKLHSQSQNEIEVQSHN--------------   681
Lu_92A3    --GGEIRRKAL------EVKEMMRNAVSDGGDGGRKGSSLIAVDEFLTAAITSTKATAADV--------------------   681
Lu_92A4    --GGEIRRKAV------EVKEMMRKAVDEG-DGGRKGSSLIAVDEFLTAAMTSTKATAADV--------------------   681
At_92A1    -VGKEIRKKAR------EVKELVRRAMVDG----VKGSSVIGLEEFLDQAMVKKVEN------------------------   681
Lu_92G2    --GEEMKRRAV------VASEELKASVRDDGDGKKKGSSAKAMDGFLADVLLADRRPVAVERVELKHGSEF----------   681
Lu_94H1    N-----------------------RVRELSEKLKGDA--DAEMDCVVDRLRSLVEGV------------------------   681
Lu_94K1    NGVG---------------EKVRRKAREMSEVMKKKG--DLEIDDVVHELLQLCRSTSVNYN-------------------   681
Si_94D1    G--------------------LRRRVEELSEKMREKG--EEEIDSLVEELVTVVRRRERSNLKSENSMKKLNVMDDGE---   681
Lu_94G1    EIK--------------------DKVRGIRDCLLEKG--DSEIDEAIDRLVHLCKTFSV----------------------   681
Lu_94G2    IIK--------------------DKAKEIKHCLLEKG--DRDIDEAIDRLVHLCKTFAE----------------------   681
Lu_94G3    EFR--------------------DKATEIQNCLLEKG--DSDIDEAIERLVHLCSNFSV----------------------   681
Lu_94G4    EFR--------------------DKAKEIKNCLVEKG--DRDIDEAIERLVHLCSEFSEPLIGEEQR--------------   681
Lu_91J1    G-Q----------------SGHRKKAAEMQTLFADTEKHESYVHDFLSNLVQALP--------------------------   681
Lu_91J2    G-Q----------------SGHREKAAEMQTLFADTEKHESYVHDFLSSLVQALD--------------------------   681
Lu_91J3    --G----------------RRHRDKAGEMKELFGHVEKHRSYVGDFLRYLEANKLN-------------------------   681
At_91A1    --G----------------KVYRENVKEMKGVFGDMDRQDRYVDSFLEYLVTNR---------------------------   681
Lu_91C2    --G----------------EGWRRAVKGAKGLFGGREKNGEMVDALVRYLTENKRNG------------------------   681
At_79B1    G------------------EKVRKNHDKWRCVLTDSGFSDGYIDKFEQNLIELVKS-------------------------   681
Lu_79B20   G------------------CSLRKNHSKWRGVVSDPDFMSSYIDKFVQNLHDLVK--------------------------   681
Lu_79B18   G------------------KEVRRNHLKLREVLGDDDLLDKYVDDFVAQLRTLLPCGDEIRHE------------------   681
Lu_79B19   G------------------KEVRRHHLKLREVLGDDHLLDKYVDDFVAQLRTLLPYGDEITRQ------------------   681
Lu_79B17   G------------------GEVRRNHLKFREVLGEKKVHDKYVDDFVVQIHDLLDA-------------------------   681
Lu_79A3    EPL----------------KSIRENQEKWKKFLLDDEAQNKYAKELVAQLKAMKISA------------------------   681
Lu_79A4    EPL----------------KSIRENQEKWKKFLLDNEAQNKYAKELVAQLKAMKNSA------------------------   681
           .......610.......620.......630.......640.......650.......660.......670.......680.
```

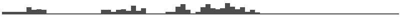

Supplement: Additional file 5 — Figure S1. Complete amino acid alignment of 137 Flax, 19 Arabidopsis and 1 Sesame UGTs. [file 1471-2164-13-175-S5.pdf]
