# Supplementary figures and images for: Phylogenomic analysis of UDP glycosyltransferase 1 multigene family in Linum usitatissimum identified genes with varied expression patterns
Source: BMC Genomics. 2012 May 8;13:175. doi: 10.1186/1471-2164-13-175 (PMC3412749; doi:10.1186/1471-2164-13-175)

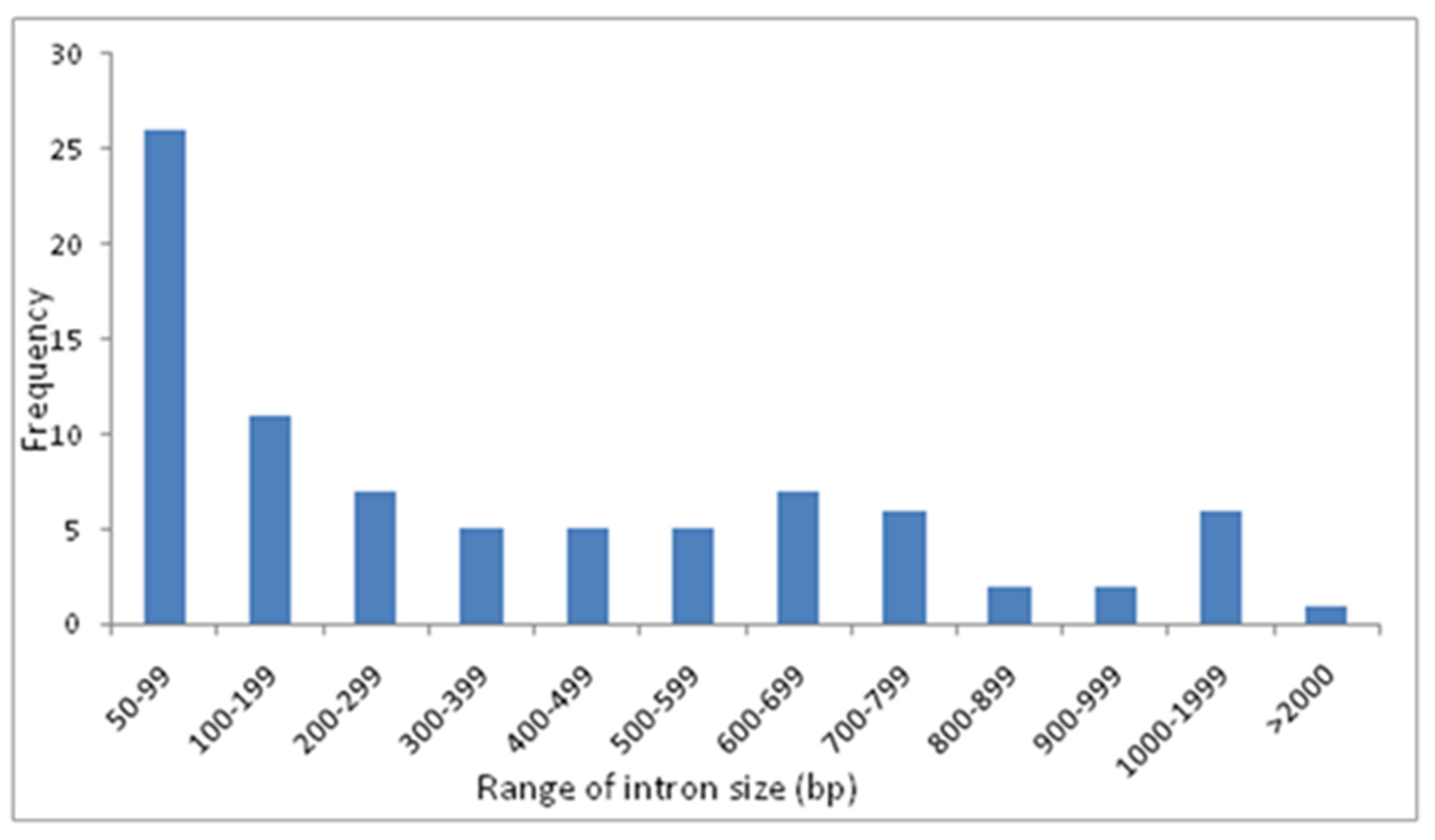

Supplement: Additional file 6 — Distribution of intron sizes in the flax UGTs. [file 1471-2164-13-175-S6.tiff]
