# Supplementary material for: Phylogenomic analysis of UDP glycosyltransferase 1 multigene family in Linum usitatissimum identified genes with varied expression patterns
Source: BMC Genomics. 2012 May 8;13:175. doi: 10.1186/1471-2164-13-175 (PMC3412749; doi:10.1186/1471-2164-13-175)
Supplement: Additional file 8 — Information about primers used to clone and sequence full length UGTs and RT-qPCR. [file 1471-2164-13-175-S8.doc]

**Additional file 8 A:** Information about primers used to clone and sequence full length flax UGTs

| **Sr. No.** | **Primer**  **name** | **Universal name** | **Phylogenetic group** | **Primer sequence (5’-3’)** | **Primer length**  **(nt)** | **Tm ^o^C** | **Amplicon size (bp)** | **Template**  **used** | **Annealing**  **temp. ^o^C** |
| --- | --- | --- | --- | --- | --- | --- | --- | --- | --- |
| 1 | S-1-F-BamHI | LuUGT71M1 | E | GGATCCATGACGCAAACGCAACTAGCT | 27 | 70 | 1476 | cDNA | 62 |
|  | S-1-R-HindIII |  |  | AAGCTTTCAACTATTTAATATAGCGCCGGATAAAAA | 36 | 68 |
| 2 | S-2-F-BamHI | LuUGT94G1 | A | GGATCCATGTTCCCGTGGTTAGCTCAC | 27 | 71 | 1693 | cDNA | 62 |
|  | S-2-R-PstI |  |  | CTGCAGCTAAACTGAAAACGTCTTACAAAGATG | 33 | 70 |
| 3 | S-4-F-BamHI | LuUGT72N1 | E | GGATCCATGCAATCCTCCGGCGC | 23 | 70 | 1476 | gDNA | 62 |
|  | S-4-R-PstI |  |  | CTGCAGTCAAGCACCTTGAGCTTTAGCG | 28 | 71 |
| 4 | S-14-F-BamHI | LuUGT85Q2 | G | GGATCCATGGGCTCTACTAACGATACAAAA | 30 | 70 | 1506 | cDNA | 62 |
|  | S-14-R-PstI |  |  | CTGCAGCTAAATCCCAACCAGTGGTTTC | 28 | 70 |
| 5 | S-16-F-XhoI | LuUGT74S1 | L | CTCGAGATGACCGTCACCGCACAATC | 26 | 71 | 1257 | cDNA | 62 |
|  | S-16-R-HindIII |  |  | AAGCTTTCAGCAGCTAAATTGGAAAGTTTCAGC | 33 | 70 |
| 6 | S-17-F-BamHI | LuUGT89B3 | B | GGATCCATGACCATGACGGTGGCC | 24 | 70 | 1431 | gDNA | 59 |
|  | S-17-R-PstI |  |  | CTGCAGTTAATCCGATGAAGAATACAATTGCAC | 33 | 70 |
| 7 | S-19-F-PstI | LuUGT72M2 | E | CTGCAGATGGAAGATGTGGTCAGTACC | 27 | 70 | 1461 | gDNA | 62 |
|  | S-19-R-EcoRI |  |  | GAATTCTCATGAACCCACCGGTTGAACC | 28 | 70 |
| 8 | S-20-F-BamHI | LuUGT712B1 | I | GGATCCATGGCGACCAAGAAGAAGAAAC | 28 | 70 | 1431 | cDNA | 62 |
|  | S-20-R-PstI |  |  | CTGCAGCTACTCACAAGCCACATCATTAG | 29 | 70 |
| 9 | GT5-F-BamHI | LuUGT85Q1 | G | GGATCCATGGGTTCGGAAGAGGGAG | 25 | 70 | 1479 | cDNA | 62 |
|  | GT5-R-HindIII |  |  | AAGCTTCTACTTGGAGTTGGGTGAAAGAAC | 30 | 70 |
| 10 | old_S-6-F-BamH1 | LuUGT72R1 | E | GGATCCATGGAAACATCAACCGTCGACG | 28 | 71 | 1506 | cDNA | 62 |
|  | old_S-6-R-HindIII |  |  | AAGCTTTTAAAATTTGGTGGCATAACGACAAGCAC | 35 | 70 |
| 11 | 15g5949-F-BamHI | LuUGT72R2 | E | GGATCCATGGAAACGTCAACCGTCGAC | 27 | 71 | 1521 | cDNA | 62 |
|  | 15g5949-R-HindIII |  |  | AAGCTTTTAAAATTTGGTGGCATAACGACAAGTAC | 35 | 70 |

**Additional file 6 B:** Information about primers used for RT-qPCR

| **Sr. No.** | **Primer**  **name** | **Universal name** | **Primer sequence (5’-3’)** | **Primer length**  **(nt)** | **Primer Conc. in reaction**  **(µM)** | **Amplicon size (bp)** | **Annealing**  **temp. ^o^C** |
| --- | --- | --- | --- | --- | --- | --- | --- |
| 1 | S-1-F-RT | LuUGT71M1 | TTACCAGACCGGCCACGGGACT | 22 | 0.4 | 144 | 60 |
|  | S-1-R-RT |  | CTACCTCCCGATCCGCCACGAC | 22 | 0.4 |
| 2 | S-2-F-RT | LuUGT94G1 | TGGGAGGGTTCGTGAGCCACTG | 22 | 0.4 | 128 | 60 |
|  | S-2-R-RT |  | CCCGGCTTCCTCGACCAGACTC | 22 | 0.4 |
| 3 | S-4-F-RT | LuUGT72N1 | GTGGGCGCGTTCGTGTCTCACT | 22 | 0.4 | 121 | 60 |
|  | S-4-R-RT |  | CCGTCAAAAGCACCGCGTTCAG | 22 | 0.4 |
| 4 | S-14-F-RT | LuUGT85Q2 | AGGGAGGTGGTGTCCGCAGGAG | 22 | 0.4 | 110 | 60 |
|  | S-14-R-RT |  | CATCGAGACGGTGACGGCCG | 20 | 0.4 |
| 5 | S-16-F-RT | LuUGT74S1 | GGCATTGCCACCATGGGAACTC | 22 | 0.1 | 109 | 60 |
|  | S-16-R-RT |  | CGCAGCTTCAAGTCCTGGCAAGT | 23 | 0.1 |
| 6 | S-17-F-RT | LuUGT89B3 | CACCGAGTTCCAACCGCAGTGA | 22 | 0.2 | 105 | 60 |
|  | S-17-R-RT |  | GTGGCGGGAAGAGGAGTGGTGA | 2 | 0.2 |
| 7 | S-19-F-RT | LuUGT72M2 | TGGTGGACGACCCATCGTTTCC | 22 | 0.4 | 100 | 60 |
|  | S-19-R-RT |  | GCTGTGTTGGATGGGGATGAAGC | 23 | 0.4 |
| 8 | S-20-F-RT | LuUGT712B1 | TCGTGCTTGATTCGTCCACTCCA | 23 | 0.4 | 100 | 60 |
|  | S-20-R-RT |  | CGTGCAAGGACCAGGAGGGTCA | 22 | 0.4 |
| 9 | GT5-F-RT | LuUGT85Q1 | CTTTGGAGACCAGCCCATTA | 20 | 0.125 | 101 | 60 |
|  | GT5-R-RT |  | CCAGCTCCTCCACATCATTT | 20 | 0.125 |
| 10 | old_S-6-F-RT | LuUGT72R1 | GTACGTCGAGTACAAACGCATC | 22 | 0.2 | 102 | 60 |
|  | old_S-6-R-RT |  | CGAAGTGCAGCGAGAGTAGAT | 21 | 0.2 |
| 11 | LuETIF5A-F | - | TGCCACATGTGAACCGTACT | 20 | 0.4 | 159 | 60 |
|  | LuETIF5A-R |  | CTTTACCCTCAGCAAATCCG | 20 | 0.4 |
