# Supplementary material for: Phylogenomic analysis of UDP glycosyltransferase 1 multigene family in Linum usitatissimum identified genes with varied expression patterns
Source: BMC Genomics. 2012 May 8;13:175. doi: 10.1186/1471-2164-13-175 (PMC3412749; doi:10.1186/1471-2164-13-175)
Supplement: Additional file 9 — Accession numbers of proteins sequences encoded by genes included in the phylogenetic analysis. [file 1471-2164-13-175-S9.doc]

**Additional file 9:** Accession numbers of Arabidopsis and Sesame (SiUGT94D1) UGT protein sequences encoded by genes included in the phylogenetic analysis

| **Sr. No.** | **Gene Name** | **GenBank Accession No.** |
| --- | --- | --- |
| 1 | AtUGT71B1 | NP_188812.1 |
| 2 | AtUGT72C1 | CAB16822.1 |
| 3 | AtUGT73C1 | NP_181213.1 |
| 4 | AtUGT74C1 | NP_180738.1 |
| 5 | AtUGT75C1 | NP_193146.1 |
| 6 | AtUGT76B1 | NP_187742.1 |
| 7 | AtUGT78D3 | NP_197205.1 |
| 8 | AtUGT79B1 | NP_200217.1 |
| 9 | AtUGT82A1 | NP_188864.1 |
| 10 | AtUGT83A1 | NP_186859.1 |
| 11 | AtUGT84A4 | NP_193285.1 |
| 12 | AtUGT85A1 | NP_193285.1 |
| 13 | AtUGT86A1 | NP_181234.1 |
| 14 | AtUGT87A1 | NP_180576.1 |
| 15 | AtUGT88A1 | NP_850597.1 |
| 16 | AtUGT89B1 | NP_177529.2 |
| 17 | AtUGT90A1 | NP_179281.3 |
| 18 | AtUGT91A1 | AAD15567.1 |
| 19 | SiUGT94D1 | BAF99027.1 |
